# Measuring viability selection from prospective cohort mortality studies: A case study in maritime pine

**DOI:** 10.1111/eva.12729

**Published:** 2019-03-18

**Authors:** Juan J. Robledo‐Arnuncio, Gregor M. Unger

**Affiliations:** ^1^ Department of Forest Ecology & Genetics INIA‐CIFOR Madrid Spain; ^2^ Escuela Internacional de Doctorado Universidad Rey Juan Carlos Móstoles Spain; ^3^ Present address: Department of Forest Genetics Federal Research and Training Centre for Forests Natural Hazards and Landscape Vienna Austria

**Keywords:** adaptive conservation management, contemporary adaptation, longitudinal study, neutrality test, *Pinus pinaster*, selection coefficient, selective mortality

## Abstract

By changing the genetic background available for selection at subsequent life stages, stage‐specific selection can define adaptive potential across the life cycle. We propose and evaluate here a neutrality test and a Bayesian method to infer stage‐specific viability selection coefficients using sequential random genotypic samples drawn from a longitudinal cohort mortality study, within a generation. The approach is suitable for investigating selective mortality in large natural or experimental cohorts of any organism in which individual tagging and tracking are unfeasible. Numerical simulation results indicate that the method can discriminate loci under strong viability selection, and provided samples are large, yield accurate estimates of the corresponding selection coefficients. Genotypic frequency changes are largely driven by sampling noise under weak selection, however, compromising inference in that case. We apply the proposed methods to analyze viability selection operating at early recruitment stages in a natural maritime pine (*Pinus pinaster* Ait.) population. We measured temporal genotypic frequency changes at 384 candidate‐gene SNP loci among seedlings sampled from the time of emergence in autumn until the summer of the following year, a period with high elimination rates. We detected five loci undergoing allele frequency changes larger than expected from stochastic mortality and sampling, with putative functions that could influence survival at early seedling stages. Our results illustrate how new statistical and sampling schemes can be used to conduct genomic scans of contemporary selection on specific life stages.

## 1 INTRODUCTION

Understanding the complex interactions between the environment, population dynamics, and adaptive evolution requires detailed analysis of selection components operating at individual stages of the life cycle. Testing for nonrandom genotype frequency changes specific to each of these stages has been the focus of classic selection component analysis, based on adult‐offspring samples (Bundgaard & Christiansen, 1972; Prout, 1971). In this context, viability (or zygotic) selection refers to differential survival of genotypes from the zygotic stage to adulthood, sexual selection to differential mating success, fecundity selection to differential offspring production, and gametic selection to prezygotic processes such as segregation distortion. In the case of viability selection, the often dramatic phenotypic changes associated with ontogeny, in addition to potentially associated changes in demography and the environment, should result in viability selection variation with age. Importantly, selective pressures operating during critical ontogenic and demographic transitions might have a disproportionate contribution to total viability selection. In the case of long‐living tree species, and many other organisms producing vast offspring cohorts, most mortality occurs during the first few months after emergence, frequently exceeding 90% during the first year (e.g., Castro, Gómez, García, Zamora, & Hódar, 1999; Vizcaíno‐Palomar, Revuelta‐Eugercios, Zavala, Alía, & González‐Martínez, 2014). Although the intensity of selection is not expected to increase rapidly with early elimination rates (Haldane, 1931), the combination of strong early seedling mortality, high inter‐individual variance in seed fecundity (Kang, Bila, Harju, & Lindgren, 2003), and substantial genetic variation at early fitness traits (e.g., Savolainen, Bokma, García‐Gil, Komulainen, & Repo, 2004) indicates ample opportunities for strong viability selection at early tree life stages (Hufford & Hamrick, 2003; Petit & Hampe, 2006; Savolainen, Pyhäjärvi, & Knürr, 2007).

Methods for detecting loci associated with viability selection during specific developmental stages are thus essential to understand the evolution of traits that are potential major contributors to total lifetime fitness and therefore important determinants of species niches and range limits under present and future climatic conditions (Donohue, 2014; Donohue, Rubio de Casas, Burghardt, Kovach, & Willis, 2010; Jackson, Betancourt, Booth, & Gray, 2009). Genomic scans for selection are a widely used tool for detecting adaptive loci, based on the comparison of observed versus expected patterns of genetic variation under particular demographic and selective models (Jensen, Foll, & Bernatchez, 2016). Several test statistics are available to identify signatures of selection from polymorphism data taken at a single point in time, such as site frequency spectra, patterns of linkage disequilibrium, or interpopulation differentiation data (reviewed in Bank, Ewing, Ferrer‐Admettla, Foll, & Jensen, 2014). These approaches are not suitable for investigating viability selection separately, however, because they are designed to detect footprints of historical selective processes, with target patterns of variation reflecting the cumulative effects of demographic and selective processes over generations, including fecundity, sexual, and gametic selection. Alternative methods use genomic time series data, tracking allele frequency trajectories over multiple generation samples, within and/or among populations (Bollback, York, & Nielsen, 2008; Feder, Kryazhimskiy, & Plotkin, 2014; Ferrer‐Admetlla, Leuenberger, Jensen, & Wegmann, 2016; Foll, Shim, & Jensen, 2015; Gompert, 2016; Malaspinas, Malaspinas, Evans, & Slatkin, 2012; Mathieson & McVean, 2013; Nishino, 2013; Schraiber, Evans, & Slatkin, 2016; Shim, Laurent, Matuszewski, Foll, & Jensen, 2016). The temporal dimension usually allows more powerful joint inference of selection coefficients and demographic parameters such as effective population size and gene flow, in the necessary disentanglement of the confounding effects of demography (versus selection) on allele frequency change across generations (Malaspinas, 2016). Even if these methods were powerful enough to infer selection over as few as two consecutive generations, however, they would still be measuring total selection and thus failing to isolate signatures of viability selection.

Genomic scans for viability selection acting on specific developmental stages require methods capable of testing allele frequency change caused by mortality during the target ontogenic period, within a single generation. For this purpose, a logistically appealing approach for long‐living tree species would consist of simultaneously sampling for genotyping several cohorts of different ages coexisting nearby within the same population. This rapid cross‐sectional sampling scheme has been used frequently in studies of neutral molecular variation across tree life stages (e.g., Alvarez‐Buylla, Chaos, Piñero, & Garay, 1996). But this strategy is questionable, at least for viability selection inference, because the ontogenetic staging of cohorts recruited at different times being necessarily asynchronous, observed allele frequency differences will be inflated by cross‐cohort differences in parental structure (e.g., due to masting and/or interannual pollination variation) and by temporal variation in selection.

Longitudinal cohort studies circumvent this problem, by sequentially sampling a target cohort of same‐age individuals multiple times as they age and die, and are more suitable for analysis of viability selection (e.g., Christiansen, Frydenberg, & Simonsen, 1978; Edwards & Heath, 1983). The few available genome‐wide analyses of selection based on longitudinal cohort studies have investigated contemporary allele frequency change caused by mortality under experimental selective regimes (Anderson, Lee, & Mitchell‐Olds, 2014; Gompert et al., 2014; Pespeni et al., 2013), after mass die‐offs caused by toxins in the wild (De Wit, Rogers‐Bennett, Kudela, & Palumbi, 2014), and in response to unknown natural selective pressures operating during critical ontogenic and demographic transitions (Bourret, Dionne, & Bernatchez, 2014) or during invasion (Monnahan, Colicchio, & Kelly, 2015). All of these studies formally tested the null neutral hypothesis that observed temporal allele frequency changes were caused by sampling and stochastic mortality alone, while two of the studies additionally estimated selection coefficients. In particular, studies where it was feasible to track and genotype exhaustively every single individual in the (small) experimental cohort did conduct selection coefficient estimation, based on observed absolute differences in survival probability between alternative homozygotes (Anderson et al., 2014; Gompert et al., 2014). The other studies focused on big natural or experimental populations, in which individual tagging and exhaustive sampling were unfeasible, so instead independent random samples were taken from the large target cohorts before and after the selective episode of interest, and no attempts were made to estimate selection coefficients from such incomplete longitudinal samples (Bourret et al., 2014; De Wit et al., 2014; Monnahan et al., 2015; Pespeni et al., 2013). The null hypothesis of genotypic‐independent mortality was still tested, either based on permutation analyses of genotypes among temporal samples (De Wit et al., 2014; Pespeni et al., 2013), homogeneity tests of genotypic proportions (Monnahan et al., 2015), or through a direct application of outlier tests designed for the analysis of historic adaptive differentiation among populations (Bourret et al., 2014). None of these studies evaluated the expected statistical performance of the employed methods with simulations, as would be required to determine their error rates and reliability (Lotterhos & Schaal, 2014).

As in the case of many tree species, natural populations of numerous other plant and animal taxa are large, with vast seedling or larval cohorts in which individual tagging and tracking may be difficult and costly. Further methods to infer viability selection from independent random samples in longitudinal cohort mortality studies would thus be broadly useful, facilitating the investigation of adaptive processes at particular ontogenetic stages and contemporary selective episodes. For instance, monitoring contemporary selection in exploited fisheries or forests during critical developmental or demographic stages could provide valuable real‐time information to orient adaptive conservation management. Measuring stage‐specific contemporary selection should also be central in studies tracking ongoing global‐change effects on the evolutionary ecology of wild or managed populations. We propose here tools to address this kind of practical questions, introducing tests of neutrality and a Bayesian scheme to estimate viability selection coefficients from independent random genotypic samples, taken sequentially from a large cohort within a generation. Using simulations, we analyze the accuracy and expected error rates of these methods under contrasting sampling and selective scenarios. Using SNP markers within a large number of candidate genes, we apply the approach to investigate viability selection operating at early recruitment stages in a relict Mediterranean maritime pine (*Pinus pinaster* Ait.) population.

## 2 MATERIAL AND METHODS

### 2.1 Demographic model

We assume a cohort of diploid individuals, of which a set of (*n*_0_, *n*_1_, …, *n*_*T*_) are sampled randomly at an initial reference time step (*t *=* *0) and at *T* subsequent time steps, with *T *≥ 1. The cohort size throughout the sampling period (*N*_0_, *N*_1_, …, *N*_*T*_) is assumed to be unknown. We assume that the sampled cohort is coetaneous, and that no new individuals join it after *t *=* *0, neither through reproduction nor through migration. The cohort is followed over time to determine whether genotypic‐dependent mortality occurs throughout one or several demographic or environmental episodes of interest, temporally delimited by the sampling intervals. For this purpose, the temporal samples are genotyped at *L* biallelic loci, yielding the **y** = $\left\{y_{\mathrm{lt}}^{11},y_{\mathrm{lt}}^{12},y_{\mathrm{lt}}^{22}\right\}$ vector of observed genotypic counts, where $y_{\mathrm{lt}}^{11}$, $y_{\mathrm{lt}}^{12}$, and $y_{\mathrm{lt}}^{22}$ are the number of individuals in the sample collected at time *t* that are homozygous for the first allele, heterozygous, and homozygous for the second allele at locus *l*, respectively.

We consider as point of reference for measuring genotypic frequency change the initial genotypic frequencies in the zygote pool originating the target cohort (**p**_0_), immediately after gamete fusion. The *N*_0_ genotypes in the cohort at *t *=* *0 can then be assumed to be multinomial draws from the initial zygote pool, while the *n*_0_ genotypes in the initial temporal sample are in turn drawn from a finite population (cohort) of size *N*_0_. This two‐step sampling is equivalent to one‐step multinomial sampling of *n*_0_ genotypes from the initial pool of genotypes, no matter whether the *n*_0_ individuals are sampled from the cohort with replacement or not (Nei & Tajima, 1981; Waples, 1989). This equivalence holds as well for subsequent samples (*t* ≥ 1) in the absence of selection. If selection operates after the initial reference sample is collected at *t *=* *0, however, then expected genotypic frequencies for *t *>* *0 will diverge from **p**_0_.

### 2.2 Quasi‐exact and Monte Carlo tests of neutrality

We propose a quasi‐exact test and a fast Monte Carlo approximation to test the null hypothesis of genotypic‐independent mortality (i.e., neutrality) in the cohort. The question is whether observed genotype frequency changes among temporal samples are greater than those expected from random mortality and sampling alone. For the *l* locus, we used the standardized variance in allele frequency for measuring the observed allele frequency change between time steps *t − *1 and *t* (Pollak, 1983; see Waples, 1989 for the two‐allele expression)

(1) $$\Delta_{\mathrm{lt}}^{\text{obs}}=\frac{{\left(f_{\mathrm{lt}}-f_{lt-1}\right)}^{2}}{(f_{\mathrm{lt}}+f_{lt-1})/2-{\left[(f_{\mathrm{lt}}+f_{lt-1})/2\right]}^{2}}$$

where $f_{\mathrm{lt}}=\left(2y_{\mathrm{lt}}^{11}+y_{\mathrm{lt}}^{12}\right)/\left(2n_{\mathrm{lt}}\right)$ and $f_{lt-1}=\left(2y_{lt-1}^{11}+y_{lt-1}^{12}\right)/\left(2n_{lt-1}\right)$. We allow for differences in sample size across loci to account for possible differences in missing data. Under the null (neutral) hypothesis, the *n*_*i*_ genotypes are multinomial draws from the initial zygote pool (with frequencies **p**_*l*0_) for any *t*. We can thus compute exactly, given **p**_*l*0,_ the cumulative probability (the *p*‐value of the test) that two independent multinomial draws of *n*_*lt*_ and *n*_*lt‐1*_ genotypes from the initial pool yield an allele frequency change equal or greater than the observed one, by adding the compound probability of all possible draws satisfying this condition, as

(2) $$\text{Pr}\left(\Delta_{\mathrm{lt}}\geq \Delta_{\mathrm{lt}}^{\text{obs}}|{\hat{p}}_{l0}\right)=\sum_{i=1}^{n_{\mathrm{lt}}}\sum_{j=1}^{\left(n_{\mathrm{lt}}-i\right)}\sum_{k=1}^{n_{lt-1}}\sum_{m=1}^{\left(n_{lt-1}-k\right)}I_{\mathrm{ijkm}}\;\cdot \;\text{Mult}\left(i,j,n_{\mathrm{lt}}-i-j|{\hat{p}}_{l0}\right)\;\cdot \text{Mult}\left(k,m,n_{lt-1}-k-m|{\hat{p}}_{l0}\right)$$

where *I*_*ijkm*_ is an indicator function having the value 1 if $\Delta_{\mathrm{lt}}^{\mathrm{ijkm}}\geq \Delta_{\mathrm{lt}}^{\text{obs}}$ and the value 0 otherwise. The $\Delta_{\mathrm{lt}}^{\mathrm{ijkm}}$ values are calculated as for $\Delta_{\mathrm{lt}}^{\text{obs}}$, but replacing *f*_*lt*_ with $f_{\mathrm{lt}}^{\prime }=\left(2i+j\right)/2n_{\mathrm{lt}}$ and *f*_*lt‐1*_ with $f_{lt-1}^{\prime }=\left(2k+m\right)/2n_{lt-1}$. The **p**_*l*0_ frequencies are unknown, so we estimated ${\hat{p}}_{l0}=\left({\hat{p}}_{l0}^{11},{\hat{p}}_{l0}^{12},{\hat{p}}_{l0}^{22}\right)$, under the null hypothesis that all temporal samples are drawn from the same initial pool and under the prior assumption that all three possible genotypes are present in the cohort (even if any of them remains undetected; see Rannala & Mountain, 1997), as

$${\hat{p}}_{l0}^{11}=\left(y_{l}^{11}+1/3\right)/\left(n_{l}+1\right),\;{\hat{p}}_{l0}^{12}=\left(y_{l}^{12}+1/3\right)/\left(n_{l}+1\right)\;\text{and}\;{\hat{p}}_{l0}^{22}=\left(y_{l}^{22}+1/3\right)/\left(n_{l}+1\right),$$

where $y_{l}^{11}$, $y_{l}^{12}$, and $y_{l}^{22}$ are the observed counts of genotypes 11, 12, and 22 in the total pooled sample, respectively, and *n*_*l*_ is the pooled sample size. We will refer to the test in Equation (2) as the quasi‐exact neutrality test, to reflect the fact that, although it is nonparametric and based on exhaustive combinatorial enumeration, it relies on the estimation of initial allele frequencies. Unlike homogeneity tests used in adult‐offspring selection component analysis (Christiansen & Frydenberg, 1973; Monnahan et al., 2015), our test does not require Hardy–Weinberg equilibrium assumptions. And in contrast to homogeneity tests specifically designed for the analysis of a sequentially sampled cohort (Christiansen et al., 1978; Edwards & Heath, 1983), our quasi‐exact test is not based on parametric asymptotic approximations, expected to break down for low genotypic counts and rare alleles (e.g., Cressie & Read, 1989).

The quasi‐exact neutrality test becomes however extremely time‐consuming for large sample sizes. Therefore, we also employed a fast approximation, by approaching the expected neutral distribution of Δ_*lt*_ with a random subsample of the possible outcomes of the compound binomial draws. In particular, we used the following Monte Carlo simulation algorithm:

1. Randomly draw the number of individuals from each genotypic class in the simulated samples at time steps *t* ($y_{\mathrm{lt}}^{\mathrm{sim}}$) and *t* − 1 ($y_{lt-1}^{\mathrm{sim}}$) from a multinomial distribution with three classes with probabilities {${\hat{p}}_{l0}^{11}$, ${\hat{p}}_{l0}^{12}$, ${\hat{p}}_{l0}^{22}$} and *n*_*lt*_ and *n*_*lt*‐1_ trials, respectively.
2. Compute the standardized allelic frequency increment in the simulated sample ($\Delta_{\mathrm{lt}}^{\mathrm{sim}}$) as in Equation (1), but using allelic frequencies calculated from the simulated genotypic counts $y_{\mathrm{lt}}^{\mathrm{sim}}$ and $y_{lt-1}^{\mathrm{sim}}$.
3. Repeat steps (1) and (2) 10,000 times and calculate the *p*‐value as the proportion of simulated replicates where $\Delta_{\mathrm{lt}}^{\mathrm{sim}}$ ≥$\Delta_{\mathrm{lt}}^{\mathrm{obs}}$.

### 2.3 Bayesian inference of selection coefficients

Under selection, expected genotypic frequencies for *t *>* *0 will diverge from **p**_0_. Denote the relative fitness of each genotype as $w_{\mathrm{lt}}^{11}$ = 1 + 2*s*_*lt*_, $w_{\mathrm{lt}}^{12}$ = 1 + 2*h*_*l*_ *s*_*lt*_ and $w_{\mathrm{lt}}^{22}$ = 1, where *s*_*lt*_ is the selection coefficient for locus *l* and time step *t*, and *h*_*l*_ is the heterozygous effect for locus *l*. The expected genotypic frequencies after selection for 0 < *t *≤* T* are then (Gillespie, 2004)

$$E\left[p_{lt+1}^{11}|p_{\mathrm{lt}},s_{\mathrm{lt}},h_{l}\right]=p_{\mathrm{lt}}^{11}w_{\mathrm{lt}}^{11}/{\overset{¯}{w}}_{\mathrm{lt}}$$

(3) $$E\left[p_{lt+1}^{12}|p_{\mathrm{lt}},s_{\mathrm{lt}},h_{l}\right]=p_{\mathrm{lt}}^{12}w_{\mathrm{lt}}^{12}/{\overset{¯}{w}}_{\mathrm{lt}}$$

$$E\left[p_{lt+1}^{22}|p_{\mathrm{lt}},s_{\mathrm{lt}},h_{l}\right]=p_{\mathrm{lt}}^{22}w_{\mathrm{lt}}^{22}/{\overset{¯}{w}}_{\mathrm{lt}}$$

where ${\overset{¯}{w}}_{\mathrm{lt}}=w_{\mathrm{lt}}^{11}p_{\mathrm{lt}}^{11}+w_{\mathrm{lt}}^{12}p_{\mathrm{lt}}^{12}+w_{\mathrm{lt}}^{22}p_{\mathrm{lt}}^{22}$ is the mean fitness of the cohort.

Given the above assumptions and the unknown initial genotypic frequencies **p**_*l*0_, selection coefficients **s**_*l*_, and dominance coefficient *h*_*l*_, the probability of observing the genotypic count **y**_*l*0_ at locus *l* in the initial sample is given by

(4) $$\text{Pr}\left(y_{l0}|p_{l0}\right)=\frac{n_{l0}}{y_{l0}^{11}!y_{l0}^{12}!y_{l0}^{22}!}{\left(p_{l0}^{11}\right)}^{y_{l0}^{11}}{\left(p_{l0}^{12}\right)}^{y_{l0}^{12}}{\left(p_{l0}^{22}\right)}^{y_{l0}^{22}},$$

while the probability of observing genotypic count **y**_*lt*_ for *t* > 0 can be written

(5) $$\text{Pr}\left(y_{\mathrm{lt}}|p_{l0},s_{lt-},h_{l}\right)=\frac{n_{\mathrm{lt}}}{y_{\mathrm{lt}}^{11}!y_{\mathrm{lt}}^{12}!y_{\mathrm{lt}}^{22}!}\;E{\left[p_{\mathrm{lt}}^{11}|p_{lt-1},s_{lt-1},h_{l}\right]}^{y_{\mathrm{lt}}^{11}}\;E{\left[p_{\mathrm{lt}}^{12}|p_{lt-1},s_{lt-1},h_{l}\right]}^{y_{\mathrm{lt}}^{12}}\;E{\left[p_{\mathrm{lt}}^{22}|p_{lt-1},s_{lt-1},h_{l}\right]}^{y_{\mathrm{lt}}^{22}}$$

where **s**_*lt*−_ is the vector of size *t* with the values of the selection coefficients for locus *l* at previous time steps (from 0 to *t *− 1). In computing $\text{Pr}(y_{\mathrm{lt}}|p_{l0},s_{lt-},h_{l})$ for the *i‐*th time step (*i *>* *0), the vector of expected genotypic frequencies are calculated sequentially from *t *=* *1 to *i* using Equation [Link], conditional on initial genotypic frequencies **p**_*l*0_, selection coefficient values at previous (*t *< *i*) time steps **s**_*lt*−_, and dominance coefficient *h*_*l*_. Note that multinomial Equation (3) assumes either that individual sampling is nondestructive (i.e., with replacement) for *t *>* *0 or that it is destructive but from a cohort of large enough size (*N*_*i*_ **>>* n*_*i*_), so that the multinomial approximates sufficiently well the actually hypergeometric sampling process.

The likelihood for locus *l* considering all time steps is then

(6) $$\text{Pr}\left(y_{l}|p_{l0},s_{l},h_{l}\right)=\text{Pr}\left(y_{l0}|p_{l0}\right)\Pi_{t=1}^{T}\text{Pr}\left(y_{\mathrm{lt}}|p_{l0},s_{lt-},h_{l}\right)$$

We employed uninformative prior distributions (*f*) for all parameters, namely a flat Dirichlet prior for the genotypic frequencies at each locus and time step, **p**_*lt*_ ~ Dir(α = 1), a uniform prior on the interval (−0.5, 10^6^) for the selection coefficient of each locus and time step (*s*_*lt*_), and a uniform prior on the interval (0, 1) for the dominance coefficient of each locus (*h*_*l*_).

Given the **y**_*l*_ vector of genotypic counts for locus *l*, the joint posterior distribution over parameter set $\Theta_{l}=\left(p_{l0},s_{l},h_{l}\right)$ is given by Bayes’ rule:

(7) $$f\left(\Theta_{l}|y_{l}\right)\propto \text{Pr}\left(y_{l}|p_{l0},s_{l},h_{l}\right)f\left(p_{l0}\right)f\;\left(s_{l}\right)f\left(h_{l}\right)$$

where the *f* functions on the right‐hand of the equation are the prior distributions. For each locus, the joint posterior distribution of Equation (4) was estimated using the MCMC algorithm described in Supporting information Appendix S1.

### 2.4 Simulation study of methods performance

Using the Monte Carlo simulations detailed in Supporting information Appendix S2, we calculated the power and Type I error rate of the quasi‐exact neutrality test (Equation (2)) and its Monte Carlo fast approximation. We also computed the expected bias, accuracy (root mean square error, RMSE), and credible interval noncoverage rate (NCR) of Bayesian estimates of selection coefficients (Equation (4)). We considered a cohort of *N* individuals, from which two temporal samples of size *n* were taken and genotyped at one locus with initial minor allele frequency MAF and selection coefficient *s*. We assumed codominance (*h* = 0.5) and investigated the effect on *s* estimates of variable levels of *N* (from 10^2^ to 10^5^), of *n* (ranging from 10^2^ to 10^4^), of *s* (0, −0.01, and −0.1), and of MAF (from 0.05 to 0.5). Exhaustive sampling scenarios (i.e., *n *= *N*) were included to assess the performance of the methods in an extreme reference setting without random sampling effects, even if the presented methods are actually inappropriate in that case, as the independent multinomial (or hypergeometric with *n *<< *N*) sampling assumption is then violated, and more direct and powerful approaches then apply (e.g., Gompert et al., 2014). All simulation and inference algorithms were coded in C++ programs. Executables of the program used for inference (sgcs) will be available from https://sites.google.com/site/jjrobledo2/software.

### 2.5 Empirical study site, field sampling, and laboratory analysis

We studied a small native population of *P. pinaster* at Fuencaliente, South‐Central Spain (38°25′N, 4°15′W), comprising around 300 adult individuals over an isolated 0.1‐km^2^ steep outcrop of quartzite, at one of the Iberian sites of the species with harshest edaphoclimatic conditions (Charco, Venturas, Gil, & Nanos, 2017; Ramírez‐Valiente & Robledo‐Arnuncio, 2014). The poor rocky soil, steep south‐facing slope (averaging 40%), high summer temperatures (with maxima occasionally exceeding 40°C), and low summer precipitation (averaging 59 mm) contribute to strong early mortality during the seed‐to‐sapling transition in the area (Charco et al., 2017). Seed dispersal by wind typically spans from May to October in Iberian populations of *P*.* pinaster*, with seedlings emerging from October to November, enhanced with the first rains after the summer drought (Juez et al., 2014; personal observations). In a preceding study at Fuencaliente, Unger, Heuertz, Vendramin, and Robledo‐Arnuncio (2016) tested the presence of exotic gene flow from conspecific plantations growing in the surroundings of the native stand, and its potential early fitness consequences. For this purpose, they took three random sequential seedling samples from a contemporary seedling cohort in the native stand, from the time of emergence in autumn until the summer of the following year. Using microsatellite markers, they found absence of seed and pollen immigration from the exotic plantations in the sampled seedling cohort. As detailed in Unger et al. (2016), seedlings were collected where found over the entire population, and the three temporal samples comprised *n*_0_ = 101 seedlings collected shortly after emergence (November 2010), *n*_1_ = 109 seedlings taken in late winter (March 2011), and *n*_2_ = 45 seedlings taken in mid‐summer (July 2011). The initial cohort size was presumably in the order of 10^5^ or more, with a study in the same population reporting over 17,000 cones (typically bearing well over ten viable seeds each) produced during a single seed dispersal season (Charco et al., 2017).

Using candidate‐gene SNP loci, we genotype here the same three temporal seedling samples to investigate whether selective mortality occurred during the first 8 months after seedling emergence at the *P*.* pinaster* population in Fuencaliente. Protocols for DNA extraction can be found in Unger et al. (2016). The 255 seedlings were genotyped with an oligo pool assay including a selection of 384 SNPs using the Illumina VeraCode platform (see table S2 in Budde et al., 2014; see also Jaramillo‐Correa et al., 2015). The used SNPs are distributed in 221 candidate genes that include drought stress candidates, genes overexpressed under abiotic stress, and functional candidates for phenology, growth and wood properties, as described in more detail in previous studies using the same assay (Budde et al., 2014; Chancerel et al., 2011; Jaramillo‐Correa et al., 2015). For each locus, we tested the null neutral hypothesis using the quasi‐exact test in Equation (2). We corrected for multiple testing by setting the expected rate of false discoveries among rejected null hypothesis at FDR = 0.05 (Benjamini & Hochberg, 1995). We proceeded to estimate selection coefficients (using Equation (4)) for loci with positive tests after FDR.

## 3 RESULTS

### 3.1 Expected accuracy of Bayesian inference of selection coefficients

**Figure 1:**
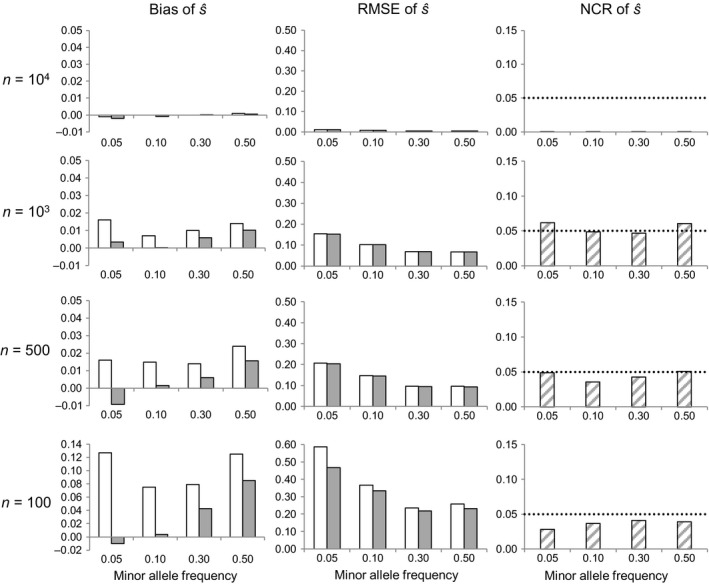
Effect of sample size (*n*) and minor allele frequency on selection coefficient estimates for a neutral locus (*s *=* *0). RMSE is the root mean square error, and NCR the noncoverage rate of 95% credible intervals (the dotted line shows the nominal 5% value). Bias and RMSE were measured with respect to the mean (white bars) or with respect to the median (gray bars). Based on 1,000 Monte Carlo replicates per scenario, assuming a cohort of size *N *=* *10,000 and a biallelic locus

No convergence problems in MCMC runs used to obtain the posterior distribution of selection coefficients (*s*) were observed for any of the simulated data sets. We present first as reference the results for a simulated cohort of size *N *= 10^4^. Assuming that the locus under study is neutral (*s* = 0), the single most important factor determining the expected errors of the selection coefficient estimates was sample size (*n*), with both the bias and RMSE of $\hat{s}$ rapidly increasing with decreasing *n* (Figure 1). Although biases were consistently lower when measured with respect to the median than with respect to the mean, the corresponding variances were similar and comparatively high, translating into very similar RMSE in general, except for the lowest sample size considered (*n *=* *100), for which the smaller RMSE of the median was more evident (white versus gray bars in Figure 1). Irrespective of whether it was calculated with respect to the median or to the mean, the RMSE of $\hat{s}$ was not affected by minor allele frequency variation between MAF = 0.3–0.5, but it increased markedly as the minor allele became more rare (for MAF = 0.1 and especially for MAF = 0.05; Figure 1). The increase in the RMSE of $\hat{s}$ with decreasing MAF was largely driven by an increment of the variance, as the bias of $\hat{s}$ either tended to actually decrease with decreasing MAF (when calculated relative to the median) or it showed a nonmonotonic relationship with MAF (when calculated relative to the mean). Overall, considering as reference minimum RMSE of $\hat{s}$ the one obtained when exhaustively sampling the entire cohort (RMSE ≤ 0.01 for *n* = *N *=* *10,000), the RMSE increased substantially for *n *< *N*, remaining at or below 0.1 only in the case of the largest random sample size considered (*n* = 1,000) with MAF ≥ 0.1, or in the case of a smaller sample (*n* = 500) with MAF ≥ 0.3. Uncertainty estimates of the method remained good in all scenarios considered, however, as the noncoverage rate (NCR) of the 95% credibility intervals (CI) of $\hat{s}$ remained around or below the nominal value of 0.05 even for the lowest sample size and MAF value (Figure 1). In other words, in the absence of selection the method would produce 95% CI for $\hat{s}$ correctly including zero as frequently as expected.

**Figure 2:**
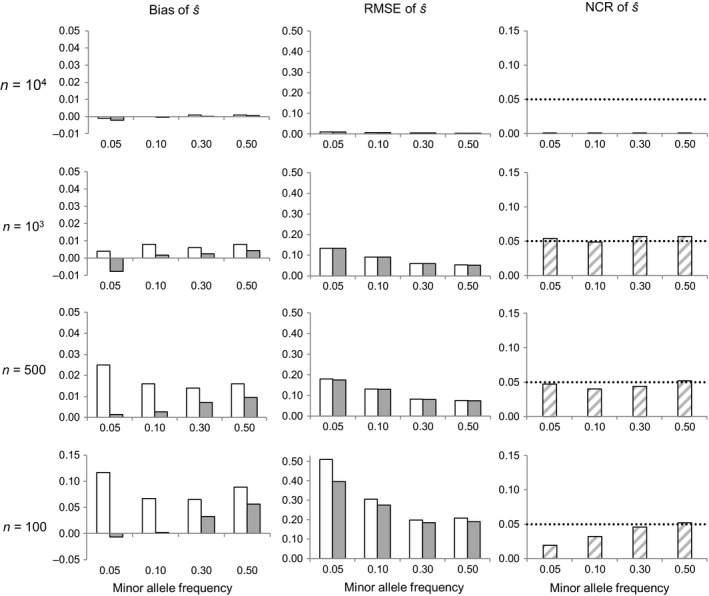
Effect of sample size (*n*) and minor allele frequency on selection coefficient estimates for a locus under strong selection (*s *= −0.1). RMSE is the root mean square error, and NCR the noncoverage rate of 95% credible intervals (the dotted line shows the nominal 5% value). Bias and RMSE were measured with respect to the mean (white bars) or with respect to the median (gray bars). Based on 1,000 Monte Carlo replicates per scenario, assuming a cohort of size *N *=* *10,000 and a locus with two codominant alleles

The simulation results revealed qualitatively similar trends in the case of a strongly selected locus (*s *= −0.1) than in the case of a neutral one, with analogous effects of sample size and minor allele frequency on estimation errors (Figure 2 versus Figure 1). Although the (absolute) bias and RMSE of $\hat{s}$ were slightly lower than in the absence of selection for all the scenarios considered, the RMSE of $\hat{s}$ was around or below its assumed absolute value (0.1) only when *n *=* *1,000 and MAF ≥ 0.1, or when *n *=* *500 and MAF ≥ 0.3 (Figure 2). The NCR remained around or below its nominal value in all cases, meaning that the method would produce 95% CI for $\hat{s}$ correctly including the value −0.1 as frequently as expected.

**Figure 3:**
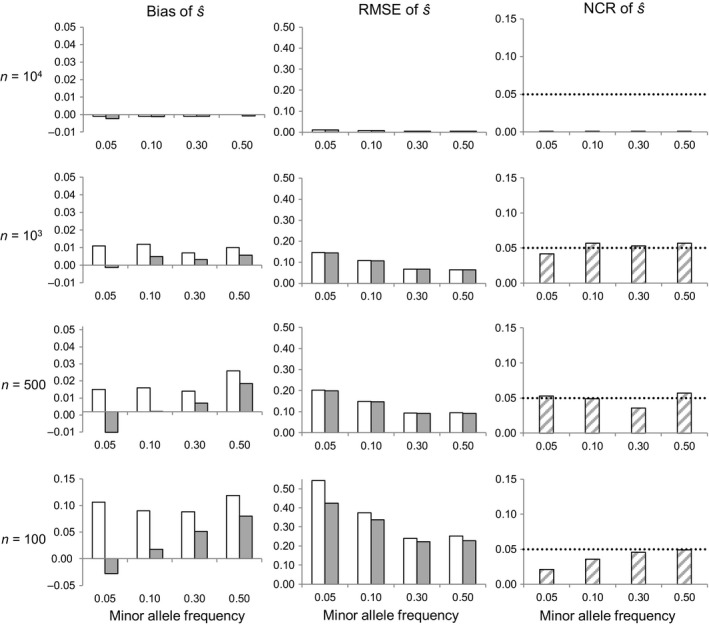
Effect of sample size (*n*) and minor allele frequency on selection coefficient estimates for a locus under weak selection (*s *= −0.01). RMSE is the root mean square error, and NCR the noncoverage rate of 95% credible intervals (the dotted line shows the nominal 5% value). Bias and RMSE were measured with respect to the mean (white bars) or with respect to the median (gray bars). Based on 1,000 Monte Carlo replicates per scenario, assuming a cohort of size *N *=* *10,000 and a locus with two codominant alleles

**Figure 4:**
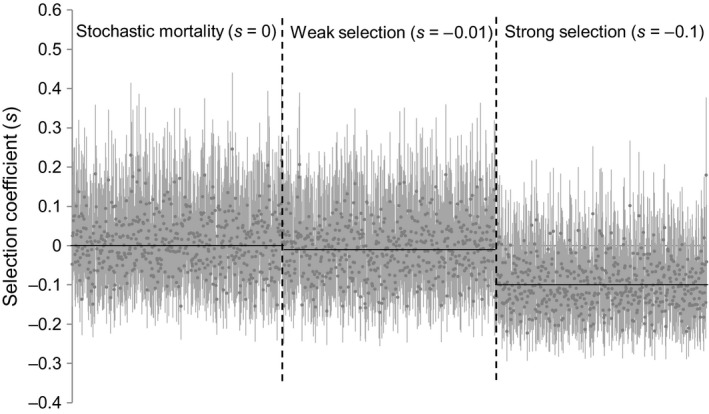
Examples of posterior probability distributions of selection coefficient estimates in a simulated cohort mortality study under different selective scenarios. Each gray point and line indicates the posterior median and 95% credibility interval for the selection coefficient of one locus in an independent Monte Carlo replicate. Horizontal black lines indicate the assumed value of the selection coefficient. Obtained assuming a large simulated cohort (*N *=* *10,000), two temporal samples of size *n *=* *1,000, and biallelic loci with minor allele frequency of 0.3

In the case of weak selection (*s *= −0.01), simulations showed again similar trends in the accuracy of $\hat{s}$ as a function of *n* and MAF as in the other selective scenarios, with absolute values for the RMSE of $\hat{s}$ generally slightly larger than those obtained for strong selection, and similar to those obtained under neutrality (Figure 3 versus Figures 1 and 2). With respect to the low assumed value (*s *= −0.01), however, the relative RMSE of $\hat{s}$ became much larger for weak than for strong selection, reaching minimum values of 50–100% when the cohort was exhaustively sampled, and exceeding 1,000% even when *n *=* *1,000 and MAF ≥ 0.1, or when *n *=* *500 and MAF ≥ 0.3 (values relative to the mean; Figure 3 shows absolute values). The NCR remained close to its nominal value in all cases (Figure 3), with wide CIs reflecting the uncertainty in *s* estimation (Figure 4).

For a given sample size *n*, the RMSE and NCR of $\hat{s}$ were weakly sensitive to the cohort size *N*, irrespective of the assumed values of *s* and MAF (Figures 1, 2, 3 versus Supporting information Figures S2‐S10). The exhaustive sampling scenarios (*N * =  *n*) were exceptional, however, as the RMSE and (especially) the NCR of $\hat{s}$ decreased relative to scenarios with the same *n* but with *n *< *N* (Figures 1, 2, 3 and Supporting information Figures S2‐S10).

### 3.2 Expected power and error rates of neutrality tests

The fast Monte Carlo approximation to the quasi‐exact neutrality test (Equation (2)) was very close in the simulated data sets, with strong *p‐*value correlation (*R* ^2^ = 0.999; Supporting information Figure S1). The simulation results reported below were obtained with the quasi‐exact test in cases with *n* ≤ 500, and via the fast approximation for *n* > 500, as the quasi‐exact test became prohibitively time‐consuming in the latter cases.

**Figure 5:**
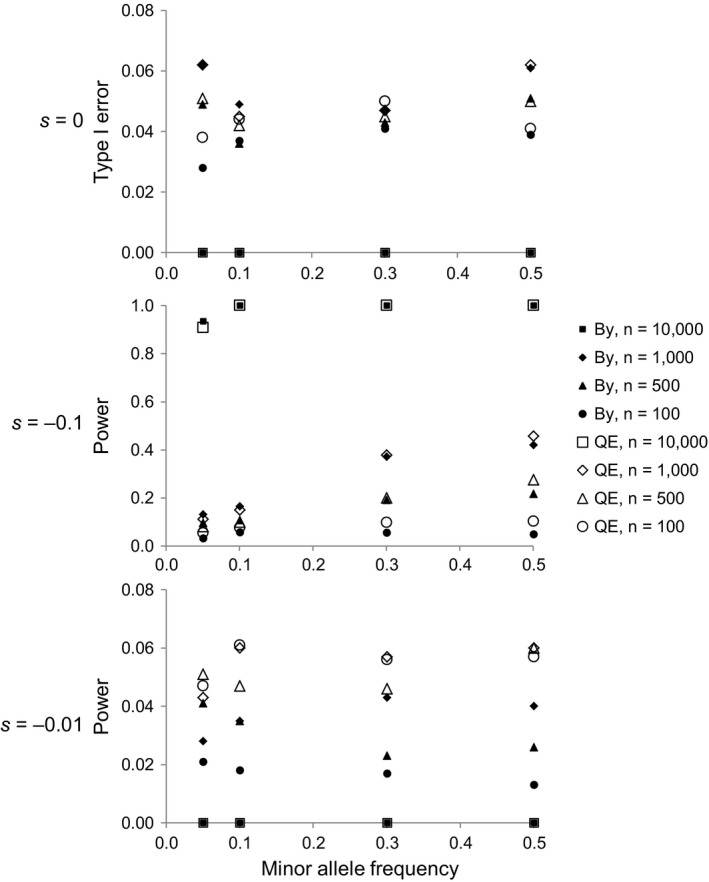
Effect of sample size and minor allele frequency on the false positive (Type I error) rate and the power of neutrality tests. The Type I error was calculated assuming a neutral locus (*s *=* *0; top panel), and the power assuming a strongly (*s* = −0.1; middle panel) or a weakly (*s* = −0.01; bottom panel) selected locus. The tests corresponded to either the quasi‐exact neutrality test in Equation (2) (“QE” white symbols) or to the proportion of times the 95% CI of Bayesian *s* estimates did not include zero (“By” black symbols). Assumed temporal sample sizes were *n *=* *100 (circles), *n* = 500 (triangles), *n* = 1,000 (diamonds) and *n* = 10,000 (squares). Based on 1,000 Monte Carlo replicates per scenario, assuming a cohort of size *N *=* *10,000 and a locus with two codominant alleles

The expected Type I error (false positive) rate of the neutrality test (assuming *s *=* *0) was zero in the extreme exhaustive sampling scenario, and ranged between 3 and 6% for the different values of sample size *n* and minor allele frequency MAF considered (Figure 5, top), that is, close to the nominal 5% value in all cases. The expected false positive rate of the neutrality test was quite similar to the NCR of 95% CI of $\hat{s}$ obtained with the Bayesian inference method when *s *=* *0, although the NCR tended to be very slightly larger for the smaller sample sizes and lower minor allele frequencies in the simulations (Figure 1 and also plotted for reference in Figure 5, top).

The power of the neutrality test was moderate‐to‐low for a strongly selected locus (*s* = −0.1), increasing with raising *n* and MAF (Figure 5, middle). Power reached up to 90–100% in the reference scenario with exhaustive sampling, decreasing to 20–50% for random samples of size *n *= 500–1,000, provided MAF ≥ 0.3, down to lows of 5–15% for the smallest samples (*n* = 100) or for larger samples when MAF ≤ 0.1 (Figure 5, middle). We compared the power of the neutrality test with that of the Bayesian approach, defined as the proportion of times the 95% CI of Bayesian *s* estimates did not include zero (when *s* ≠ 0). The power of the Bayesian approach also increased with *n* and MAF, with the neutrality test having slightly greater power in general, especially for the smallest sample size, but slightly lower power for larger samples with MAF ≤ 0.1 (Figure 5, middle).

The neutrality test most often failed to reject the null neutral hypothesis for loci under weak selection (*s* = −0.01), with a very low power of 4–6% for all simulated values of *n* and MAF (Figure 5, bottom), of the same order than the expected false positive rate for neutral loci (Figure 5, top). Power was even zero in the theoretical exhaustive sampling scenario, suggesting that the violation of the independent sampling assumption precluded the statistical detection of weak selection. The power of the Bayesian approach was slightly below that of the neutrality test for all simulated weak selection scenarios (Figure 5, bottom).

For a given sample size *n*, changes in the cohort size *N* did not substantially alter the power and false positive rate of either the neutrality test or the Bayesian approach, except for the extreme reference scenarios with exhaustive sampling (*N *= *n*), when the model assumptions are violated and power generally dropped relative to cases with the same *n* but *N *>* n*, while the expected false positive rate remained invariably close to nominal levels (Figure 5 versus Supporting information Figures S11‐S13).

### 3.3 Genomic analysis of early selection in *Pinus pinaster*

**Figure 6:**
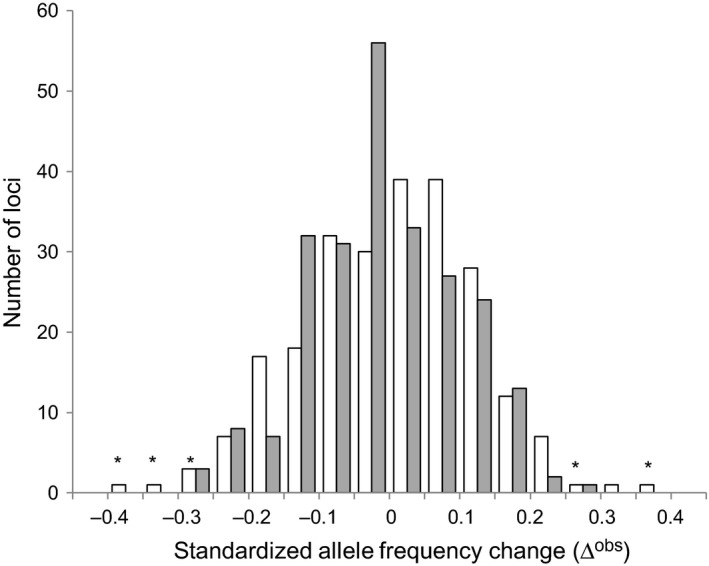
Distribution of standardized temporal allele frequency changes observed at 237 polymorphic SNP loci in a *Pinus pinaster* seedling cohort mortality study in Fuencaliente (Spain). The two sampling periods were November 2010–March 2011 (white bars) and March–July 2011 (gray bars). Changes larger than expected (*p* < 0.05 after FDR correction) from random mortality and sampling are marked with asterisks

The 384‐plex SNP assay produced reliable seedling genotypes at 356 loci, of which 302 were polymorphic. The genomic analysis of selection was conducted on 237 (78%) polymorphic loci having MAF > 0.05, which exhibited a substantial range of temporal allele frequency change, somewhat wider during the first (winter) than during the second (spring‐summer) target periods (Figure 6). The allele frequency change was not larger than expected by random mortality and sampling for most loci, as indicated by the quasi‐exact neutrality test (Figure 6). In particular, there was no significant evidence of genotypic‐dependent mortality during the second study period for any locus. During the first period, however, allele frequencies at 5 loci showed temporal changes significantly larger than expected from random mortality and sampling (Figure 6 and Table 1). The same set of loci was identified with the quasi‐exact test and the fast Monte Carlo approximation (results not shown). Bayesian estimates of selection coefficients were significantly different from zero for the five loci that exhibited exceptional allele frequency change, with the strength of selection, based on median point estimates, ranging from 0.29 to 0.47 (Table 1). The loci potentially under selection are found in genes related with water stress response (*m650*), abiotic stress responses (*m1115*), disease resistance (*m1496*), or more general functions such as cellular signaling (*m102*) and protein assembly (*m698*).

**Table 1:** Identity, annotation, and Bayesian selection coefficient estimates ($\hat{s}$) for five SNPs exhibiting significant temporal allele frequency change in the *Pinus pinaster* prospective cohort mortality study

EST contig or amplicon	SNP name	Annotation	SNP motif^a^	MAF^b^	$\Delta^{\mathrm{obs}}$ (*p*‐value)^c^	Median $\hat{s}$ (95% CI)
2_10052_01	*m1496*	Putative TIR/NBS/LRR disease resistance protein [*Arabidopsis thaliana*]	**A**/C	0.384	0.268 (0.014)	0.343 (0.202, 0.417)
2_4724_01	*m102*	Putative serine/threonine protein kinase	C/**G**	0.152	0.372 (0.002)	0.373 (0.251, 0.432)
CT_2227	*m650*	Putative ADP‐ribosylation factor	A/**G**	0.485	−0.307 (0.001)	−0.337 (−0.417, −0.181)
CT_3813	*m698*	Nascent polypeptide‐associated complex (NAC)	A/**G**	0.429	−0.260 (0.014)	−0.294 (−0.398, −0.094)
UMN_5272_01	*m1115*	6‐phosphogluconate dehydrogenase (6pgdB)	A/**G**	0.146	−0.383 (0.001)	−0.465 (−0.499, −0.290)

a The allele increasing in frequency appears in bold.

b Minor allele frequency.

c Observed allele frequency increment (*p*‐value provided by quasi‐exact neutrality test after FDR correction).

## 4 DISCUSSION

Motivated by the need to quantify contemporary selection acting on specific ontogenetic stages, we have proposed and evaluated a neutrality test and a Bayesian method to infer selection coefficients using random genotypic samples drawn from a longitudinal cohort mortality study. The approach should be of interest for researchers investigating selective mortality in large natural or experimental cohorts of any organism in which individual tagging and tracking are unfeasible. We have applied the proposed methods in a natural maritime pine seedling cohort, detecting five loci undergoing exceptional allele frequency change during a short early establishment stage with high elimination rates.

### 4.1 Measuring selection from cohort mortality studies

Our model represents a first attempt to explicitly estimate contemporary selection coefficients from random genotypic samples taken sequentially from a large cohort mortality study, within a generation. Even if genetic cohort studies are free from genetic drift effects complicating selection inference from multigeneration time series (e.g., Feder et al., 2014), our simulation analysis shows for the first time that distinguishing within‐generation allele frequency changes caused by selective mortality from the stochastic noise produced by sampling and genotype‐independent mortality remains an inherently difficult statistical exercise. A reassuring aspect of the results, however, is that the method does not tend to mistake neutral loci as being under selection more frequently than expected, since the false positive rate of the neutrality test and the NCR of Bayesian *s* estimates (when *s* = 0) remain close to nominal values, independently of sample size and minor allele frequency. Results are also promising in terms of the power to discriminate loci under strong selection, which should be achievable with moderate or even high confidence by sufficiently increasing the sample size, provided the target locus is not close to fixation (Figure 5, middle). Moreover, median‐based point estimates of the selection coefficient obtained with the Bayesian method can be expected to be minimally biased for strongly selected loci, although, as a consequence of their large variance, large samples will be necessary to reduce the relative RMSE below 100% (Figure 2). Prospects for detecting and quantifying weak selection are bleaker, as the probability to discriminate a weakly selected locus is similar to the probability of mistaking a neutral locus as nonneutral (Figure 5), and as the RMSE of Bayesian *s* estimates become very large relative to small *s* values even for large samples (Figure 3). This is because observed allele frequency changes in the cohort are largely driven by sampling noise under weak selection, an intrinsic problem that seems difficult to circumvent (as already noted by Christiansen et al., 1978) and is shared with methods based on multigenerational temporal genotypic samples (Feder et al., 2014; Gompert, 2016).

### 4.2 Model assumptions and perspectives

Unlike statistical methods to infer selection from multigeneration genetic time series data, our model does not require assumptions on (or joint estimation of) historical demographic and reproductive processes that may erase, alter, or mimic footprints of selection on genotypic frequency change across generations, such as fluctuations in effective population size and gene flow among divergent populations. Nor does the model assume Hardy–Weinberg equilibrium within the sampled cohort, as genotypic frequencies are estimated directly, rather than based on expected random‐mating proportions. The method relies however on some potentially limiting assumptions that should be noted. First, samples taken from the target cohort are assumed to be free of immigrants and recruits newly established after the initial sampling step, either because they do not exist or because they can be discriminated during sampling (as they could represent a confounding source of temporal genotypic frequency change). This requirement will be easily met in experimental studies, but also in natural populations of many organisms, such as plant species for which both seed dispersal and germination occur in discrete synchronous periods, or animal species with synchronous reproduction and temporally coherent juvenile cohorts (e.g., anadromous fishes). Otherwise, special caution should be taken, for instance when studying seedling cohorts in plants with spread germination periods, since genetically determined emergence phenology (Donohue, 2005) might inflate temporal variation in genotypic frequencies. The model also assumes a cohort of same‐age individuals, considering as reference population the initial zygotic pool that originated such cohort. If multiple age classes were present, then the total cohort itself (at the time of the initial sample) could be considered as reference population, and our methods should presumably be robust to this reference change as long as the cohort is large.

Another assumption of the model is that sampling is random, that is, that the probability of sampling a genotype does only depend on its frequency in the cohort at the time of collection. This assumption could be violated if both individuals and genotypes are spatially structured in the study area and sampling is biased toward high‐density groups, especially if there are temporal changes in local density (e.g., due to stochastic plant mortality or animal movement). To avoid this problem, individual sampling should be proportional to local abundance at the time of sampling. The random sampling assumption would also be violated if there were genetically determined phenotypic differences among individuals that could bias sampling between temporal samples (e.g., sex‐biased sampling between life stages in anadromous salmons; Bourret et al., 2014).

If in practice individuals were sampled without replacement, the model would then also be assuming that the cohort under study is large, so that the distribution of observed genotypic counts after selection is approximately multinomial, remaining therefore largely independent of the unknown cohort size *N*. It would be possible to drop the large cohort size assumption in cases where sampling is destructive, by assuming a hypergeometric distribution instead of a multinomial in Equation (3). The latter would require however joint estimation of the unknown cohort size ***N***, which in practice results in important numerical optimization problems, while yielding very similar estimates of the parameters of interest (***s*** and *h*) and largely uninformative posterior distributions of ***N***, which suggests that the multinomial approximation under destructive sampling is good unless ***N*** is small (results not shown). The presented simulation results showed that exhaustively sampling a small cohort, which violates the independent sampling assumption, tends to hamper the statistical detection of selection (tests become nonsignificant), especially for weak selection, even if the RMSE of the (nonsignificantly different from zero) selection coefficient estimates actually decrease. This is not, however, a drawback of our random‐sampling oriented methods, as they are not intended for studying a cohort small enough that every individual could be tracked, in which case more straightforward and powerful approaches should be employed (e.g., Anderson et al., 2014; Gompert et al., 2014).

In summary, as guidance for empirical studies, our approach should work best when large independent random genotypic samples are sequentially taken from a vast cohort of same‐age individuals, in which immigrants or new recruits arriving after the time of the first temporal sample either do not exist or can be easily discriminated. Ideally, the cohort should consist of same‐age individuals, but the presence of multiple age classes is unlikely to compromise inference if the cohort is large. As a rule of thumb, loci under strong selection should be detectable with acceptable power, and the corresponding selection coefficients estimated with acceptable accuracy, for sample sizes in the order of 10^3^ genotypes randomly taken from a cohort of size in the order of 10^4^ individuals or more.

Several further tests and developments of the presented model are possible. We did not investigate the effect of the number of temporal samples on selection estimates. The model assumes two or more temporal samples, collected before and after one or more consecutive periods with potentially variable selection. Although separate selection coefficients are estimated for each period, they are not independent, because the expected genotypic frequencies at the end of each time interval are calculated conditional on initial frequencies and selection coefficients during that and preceding periods. Preliminary simulation results (not shown) indicate that adding temporal samples does not significantly affect the accuracy of per‐period selection coefficient estimates, assuming codominance during simulations and inference. Future studies may address how unknown and contrasting heterozygous effects, and their joint estimation, affect selection estimates, an underexplored issue in most statistical methods inferring selection, including those based on time series data (but see Teshima, Coop, & Przeworski, 2006; Shim et al., 2016). Although estimating unknown heterozygous effects should complicate inference of selection coefficients (Shim et al., 2016), adding temporal samples might improve their joint estimation if the heterozygous effect remained approximately constant across sampling periods.

Our model neither makes assumptions nor provides information on whether correlated selection among loci occurs or not (e.g., due to genetic linkage or epistasis). Estimated selection coefficients represent differences in expected single‐locus marginal fitness, that is, difference in relative fitness between the alternative homozygotes in a locus, averaged over genomic backgrounds (Ewens, 2004; Gompert et al., 2014). The ability of the model to detect selection will thus depend on the size of expected locus‐specific effects, including direct (for causal genetic variants) and/or indirect (for loci correlated with causal variants) effects. The method can be expected to have rather low power in the case of polygenic selection acting on many small‐effect loci, as is the norm in single‐locus‐based genomic scan methods. In that case, attempting the explicit estimation of selection coefficients may not prove a rewarding statistical exercise, and alternative multivariate approaches testing nonrandom temporal covariation in allele frequencies among loci might be more helpful for detecting traces of selection (e.g., Bourret et al., 2014; Laporte et al., 2016).

### 4.3 Contemporary selection on maritime pine recruits

We investigated for the first time whether genotypically based selection could determine nonrandom mortality during a critical early developmental stage in a tree species. In line with the model assumptions, seedling emergence is expected to be largely synchronous in the species, and samples were obtained over the entire population area, proportionally to local abundance and nondestructively whenever possible (Unger et al., 2016). Despite the vast cohort size at the initial seed‐crop stage (also in line with the model assumptions), and even if collected seedlings represented a considerable portion of surviving individuals at the time of sampling (with virtually exhaustive sampling during the last period), the low emergence rates and strong early mortality in the area did not allow large samples, which according to the simulations likely reduced substantially the power of the selection analysis, though without increasing the false positive rate. Still, consistently with recent genomic scans of selection based on prospective cohort studies (De Wit et al., 2014; Gompert et al., 2014; Pespeni et al., 2013), our results provide evidences of selective elimination during particular life stages inducing significant allele frequency changes within a single generation. Selection at early life stages can define adaptive potential across the life cycle, as it changes the genetic background available for selection at subsequent life stages, especially in novel environments or extreme climatic events, and when pleiotropy is present (Donohue, 2014).

We used candidate‐gene marker loci, so positive tests must necessarily correspond to putatively functional SNPs. It is however noteworthy that three of the five identified loci potentially under selection during the first 4 months after seedling emergence have been related with functions that could influence survival precisely at early stages. In particular, the putative ADP‐ribosylation factor (SNP *m650*; Table 1) has shown altered expression levels in *P. pinaster* seedlings subject to experimental water deficit (Dubos et al., 2003). The SNP *m102* encodes proteins related to the protein‐kinase family, associated with general cellular signaling functions increased during pine seedling development (Ávila, Pérez‐Rodríguez, & Cánovas, 2006). And the putative NAC transcription factor encoded by *m698* belongs to a family apparently involved in abiotic stress responses of *P. pinaster* seedlings (Pascual, Cánovas, & Ávila, 2015). Of the two other identified loci, the putative protein encoded by *m1496* is related to the TIR/NBS/LRR disease resistance protein family, found in *Arabidopsis* and other plant genomes including pines (Meyers, Morgante, & Michelmore, 2002), and the 6‐phosphogluconate dehydrogenase protein‐coding gene (*m1115*) is associated with general abiotic stress responses in several model plant species (Hou, Huang, Yu, & Zhang, 2007).

The estimated average absolute strength of selection experienced by the five loci over the 4‐month period was rather high (0.362), though of the same order than the one estimated for loci undergoing exceptional temporal allele frequency change in another short‐term cohort mortality study, which focused on rapid adaptation of stick insects to a sudden host shift (Gompert et al., 2014). These values should obviously not be regarded as unbiased estimates of the average strength of genome‐wide selection, since they correspond to a small subset of sampled loci with the strongest evidence of selection, and estimates of locus‐specific size effects based on crossing significance thresholds are expected to be upwardly biased (Göring, Terwilliger, & Blangero, 2001; Ioannidis, 2008; Williams & Haines, 2011). Moreover, the observed exceptional allele frequency changes were probably determined by both selective mortality and sampling noise, with their relative contributions being unknown (Gompert et al., 2014).

Apart from statistical and stochastic factors, two main biological reasons could help explain the significant signals of strong stage‐specific selection observed in our field cohort study. First, drought stress is a major driver of pine early seedling mortality in the Iberian Peninsula (Castro, Zamora, Hódar, & Gómez, 2004; Vizcaíno‐Palomar et al., 2014), and recent mortality could be genotype‐dependent because the relict *P. pinaster* population might be currently undergoing rapid genetic adaptation to new drier conditions. Recent climate change is actually resulting in a higher warming rate across Spain than the global average, accompanied by reductions in relative humidity and rainfall (Vicente‐Serrano, Rodríguez‐Camino, Domínguez‐Castro, El Kenawy, & Azorín‐Molina, 2017). The potential selective pressures exerted by ongoing climate change would be greatly enhanced by the human‐mediated severe range contraction suffered by the studied population in historic times (Charco et al., 2017), which has enforced a strong habitat shift, from the nonmarginal historic one to which the current population could still be genetically adapted, to the only recently occupied extremely marginal microenvironment. An alternative biological explanation consistent with our strong selection estimates is that viability selection (measured by survival) tends to be stronger when measured over shorter periods, which could result from selection over longer time intervals tempering bursts of strong selection, via reversal or stasis periods (Hoekstra et al., 2001).

Beyond increasing sample sizes for enhanced statistical power, further insights could be gained by replicating our study on different cohorts, newly established over subsequent seed dispersal seasons. It would then be possible to test whether the same set of loci tend to exhibit nonrandom temporal allele frequency changes for cohorts established under similar seasonal environmental conditions. This would help supporting the true association of detected loci with selective mortality under a particular environment (Chanock et al., 2007). If in addition reverse locus‐specific allelic frequency changes were consistently detected in cohorts established under contrasting environmental conditions (e.g., dry versus wet), it would suggest that fluctuating selection is acting at early developmental stages, a process potentially contributing to the high within‐population genetic variation of trees (Yeaman & Jarvis, 2006). It would be particularly interesting to track cohorts established under the typically rare combination of favorable environmental conditions allowing a successful transition to the sapling stage (Jackson et al., 2009), as it may reveal the otherwise hidden selective patterns that are mostly shaping the adaptive genetic variation of individuals reaching reproduction. Finally, by extending the tracking period of successfully recruited sapling cohorts, it would be possible to study which life stages are under the most intense natural selection, whether pleiotropy is present across life stages, and whether the strength of selection changes with the temporal scale of measurement (Donohue, 2014; Hoekstra et al., 2001). The methods presented here should provide a useful tool for addressing these fundamental questions in many species with large offspring cohorts and may open the way for further statistical advances for genomic analyses of selection on cohort mortality studies.

## CONFLICT OF INTEREST

None declared.

## DATA ARCHIVING STATEMENT

Data for this study are available at the Dryad Digital Repository: https://doi.org/10.5061/dryad.3p5n8bj.
