# Supplementary material for: Measuring viability selection from prospective cohort mortality studies: A case study in maritime pine
Source: Evol Appl. 2019 Mar 18;12(5):863–77. doi: 10.1111/eva.12729 (PMC6503825; doi:10.1111/eva.12729)
Supplement: Supplementary file 1 [file EVA-12-863-s001.docx]

**Supporting Information for:**

**Measuring Viability Selection from**

**Prospective Cohort Mortality Studies: a Case Study in Maritime Pine**

Juan J. Robledo-Arnuncio & Gregor M. Unger

**Table of Contents:**

| **Appendix S1 *MCMC algorithm*** | Page 2 |
| --- | --- |
| **Appendix S2 *Details of Monte Carlo analysis of method performance*** | Page 4 |
| **Figure S1 *Scatter plot of p-values obtained with the quasi-exact neutrality test***  ***and the Monte Carlo fast approximation*** | Page 6 |
| **Figure S2 Effect of sample size and minor allele frequency on selection coefficient**  **estimates for a neutral locus and a cohort of size *N* = 100,000** | Page 7 |
| **Figure S3 Effect of sample size and minor allele frequency on selection coefficient**  **estimates for a locus under strong selection and a cohort of size *N* = 100,000** | Page 8 |
| **Figure S4 Effect of sample size and minor allele frequency on selection coefficient**  **estimates for a locus under weak selection and a cohort of size *N* = 100,000** | Page 9 |
| **Figure S5 Effect of sample size and minor allele frequency on selection coefficient**  **estimates for a neutral locus and a cohort of size *N* = 1,000** | Page 10 |
| **Figure S6 Effect of sample size and minor allele frequency on selection coefficient**  **estimates for a locus under strong selection and a cohort of size *N* = 1,000** | Page 11 |
| **Figure S7 Effect of sample size and minor allele frequency on selection coefficient**  **estimates for a locus under weak selection and a cohort of size *N* = 1,000** | Page 12 |
| **Figure S8 Effect of sample size and minor allele frequency on selection coefficient**  **estimates for a neutral locus and a cohort of size *N* = 500** | Page 13 |
| **Figure S9 Effect of sample size and minor allele frequency on selection coefficient**  **estimates for a locus under strong selection and a cohort of size *N* = 500** | Page 14 |
| **Figure S10 Effect of sample size and minor allele frequency on selection coefficient**  **estimates for a locus under weak selection and a cohort of size *N* = 500** | Page 15 |
| **Figure S11 Effect of sample size and minor allele frequency on the false positive**  **rate and the power of neutrality tests for a cohort of size *N* = 100,000** | Page 16 |
| **Figure S12 Effect of sample size and minor allele frequency on the false positive**  **rate and the power of neutrality tests for a cohort of size *N* = 1,000** | Page 17 |
| **Figure S13 Effect of sample size and minor allele frequency on the false positive**  **rate and the power of neutrality tests for a cohort of size *N* = 500** | Page 18 |

**Appendix S1** *MCMC algorithm*

We used the Metropolis-Hastings (MH) algorithm (Metropolis, Rosenbluth, Rosenbluth, Teller, & Teller, 1953; Hastings, 1970) to approximate numerically the posterior probability density of model parameters. The implementation of the algorithm involved three steps at each MH iteration, with a different parameter vector being potentially modified at each step, as described below. Initial values for the genotypic frequencies in the initial temporal sample (**p_0_**) were set at their observed values in the sample. Initial values for **s** and **h** were set at zero (neutrality) and 0.5 (co-dominance), respectively.

*Updating selection coefficients (***s***_l_)*

For each locus *l*, we updated selection coefficients of all sampling time-steps at every MH iteration, one time-step at a time. For each sampling time step *t*, we propose a new value $s_{lt}^{'}$ = *u*, where *u*~ *U*[max(−0.5, *s_lt_* − *e_s_*), min(10^6^, *s_lt_* + *e_s_*)] and *e_s_* is some incremental value. Let $\mathbf{s}_{l}^{'}$ be the vector of selection coefficients **s***_l_* with element *s_lt_* replaced with $s_{lt}^{'}$. This move is accepted with probability

$\alpha(\mathbf{s}_{l}^{'}|\mathbf{s}_{l})=\min\left(1,\frac{\Pr(\mathbf{y}_{l}|\mathbf{p}_{l0},\mathbf{s}_{l}^{'},h_{l})f(\mathbf{s}_{l}^{'})q(\mathbf{s},\mathbf{s}_{l}^{'})}{\Pr(\mathbf{y}_{l}|\mathbf{p}_{l0},\mathbf{s}_{l},h_{l})f(\mathbf{s}_{l})q(\mathbf{s}_{l}^{'},\mathbf{s}_{l})}\right)$,

where $q(\mathbf{s},\mathbf{s}_{l}^{'})$ is the uniform density described above and $q(\mathbf{s}_{l}^{'},\mathbf{s}_{l})$ is the reverse move uniform density *U*[max(−0.5, $s_{lt}^{'}$ − *e_s_*), min(10^6^, $s_{lt}^{'}$ + *e_s_*)]. Note that calculating $\Pr(\mathbf{y}_{l}|\mathbf{p}_{l0},\mathbf{s}_{l}^{'},h_{l})$ requires sequentially recalculating expected genotypic frequencies at locus *l* from time step *t* + 1 to *T* (using eqn 3 in the main text), conditional on the proposed $s_{lt}^{'}$.

*Updating the dominance coefficient (h_l_)*

We updated the dominance coefficient *h_l_* of locus *l* at every MH iteration. We propose a new value $h_{l}^{'}$ = *u*, where *u*~ *U*[max(0, *h_l_* − *e_h_*), min(1, *h_l_* + *e_h_*)] and *e_h_* is some incremental value. This move is accepted with probability

$\alpha(h_{l}^{'}|h_{l})=\min\left(1,\frac{\Pr(\mathbf{y}_{l}|\mathbf{p}_{l0},\mathbf{s}_{l},h_{l}^{'})f(h_{l}^{'})q(h_{l},h_{l}^{'})}{\Pr(\mathbf{y}_{l}|\mathbf{p}_{l0},\mathbf{s}_{l},h_{l})f(h_{l})q(h_{l}^{'},h_{l})}\right)$,

where $q(h_{l},h_{l}^{'})$ is the uniform density described above and $q(h_{l}^{'},h_{l})$ is the reverse move uniform density *U*[max(0, $h_{l}^{'}$ − *e_h_*), min(1, $h_{l}^{'}$ + *e_h_*)]. Note that calculating $\Pr(\mathbf{y}_{l}|\mathbf{p}_{l0},\mathbf{s}_{l},h_{l}^{'})$ requires sequentially recalculating expected genotypic frequencies at locus *l* from time step 1 to *T* (using eqn 3 in the main text), conditional on the proposed $h_{l}^{'}$.

*Updating initial genotypic frequencies* *(***p***_l_*_0_*)*

We updated the initial sampling time-step genotypic frequencies **p***_l_*_0_ of locus *l* at every MH iteration. We randomly choose two of the three possible genotypes, *a* and *b*, and propose $p'_{l0}^{a}$= *u*, where *u* ~ *U* [max(0, $p_{l0}^{a}$ − *e_p_*), min($p_{l0}^{a}$ + *e_p_*, $p_{l0}^{a}$ + $p_{l0}^{b}$)] and *e_p_* is some incremental value. We then set $p'_{l0}^{b}$= $p_{l0}^{a}$ + $p_{l0}^{b}$ −$p'_{l0}^{a}$ and accept the move with probability

$\alpha(\mathbf{p}_{l0}^{'}|\mathbf{p}_{l0})=\min\left(1,\frac{\Pr(\mathbf{y}_{l}|\mathbf{p}_{l0}^{'},\mathbf{s}_{l},h_{l})f(\mathbf{p}_{l0}^{'})q(\mathbf{p}_{l0},\mathbf{p}_{l0}^{'})}{\Pr(\mathbf{y}_{l}|\mathbf{p}_{l0},\mathbf{s}_{l},h_{l})f(\mathbf{p}_{l0})q(\mathbf{p}_{l0}^{'},\mathbf{p}_{l0})}\right)$,

where $\mathbf{p}_{l0}^{'}$ is the vector **p***_l_*_0_ with elements $p_{l0}^{a}$ and $p_{l0}^{b}$ replaced with $p'_{l0}^{a}$ and $p'_{l0}^{b}$, $q(\mathbf{p}_{l0},\mathbf{p}_{l0}^{'})$ is the uniform density described above, and $q(\mathbf{p}_{l0}^{'},\mathbf{p}_{l0})$ is the reverse move uniform density *U* [max(0, $p'_{l0}^{a}$ − *e_p_*), min($p'_{l0}^{a}$ + *e_p_*, $p'_{l0}^{a}$ + $p'_{l0}^{b}$)]. Note that calculating $\Pr(\mathbf{y}_{l}|\mathbf{p}_{l0}^{'},\mathbf{s}_{l},h_{l})$ requires sequentially recalculating expected genotypic frequencies at locus *l* from time step 1 to *T* (using eqn 3 in the main text), conditional on the proposed $p'_{l0}^{a}$ and $p'_{l0}^{b}$.

Pilot runs were used to tune up all proposal distributions to obtain acceptance rates between 40−50%, after which the MCMC algorithm was run for 260,000 iterations, discarding the first 10,000 as burn-in and thinning the remaining ones to every 25th, yielding a final sample of 1,000 observations.

**References**

Hastings, W. K. (1970). Monte carlo sampling methods using Markov chains and their applications. *Biometrika*, *57*(1), 97–109. doi:10.1093/biomet/57.1.97

Metropolis, N., Rosenbluth, A. W., Rosenbluth, M. N., Teller, A. H., & Teller, E. (1953). Equation of state calculations by fast computing machines. *The Journal of Chemical Physics*, *21*(6), 1087–1092. doi:http://dx.doi.org/10.1063/1.1699114

**Appendix S2** *Details of* *Monte Carlo analysis of method performance*

Given the assumed population size *N*, selection coefficient *s*, dominance coefficient *h*, sample size *n*, and minor allele frequency *MAF* (with alleles 1 and 2 having population frequencies *MAF* and 1− *MAF*, respectively), each independent replicate of the stochastic simulations used to test the model performance involved the following six steps:

1. Generate the genotype of each of the *N* individuals at *t*= 0 in the population with two independent draws from a binomial distribution with probability *MAF*.
2. Randomly sample *n* individuals at *t* = 0 from the population of *N* individuals (or sample all *N* individuals in the exhaustive sampling scenarios).
3. For each individual in the population, draw a uniform random variate *r* on [0, 1]. The individual survives to time-step *t*= 1 if either: (a) it’s homozygous for the second allele; (b) it’s heterozygous and *r*< 1 + 2*hs*; or (c) it’s homozygous for the first allele and *r*< 1 + 2*s*.
4. Randomly sample *n* individuals at t = 1 from the surviving individuals in the population (or sample all surviving individuals in the exhaustive sampling scenarios).
5. Given the two temporal genotypic samples of size *n*, estimate posterior distributions of parameters $\mathbf{\Theta}$, with corresponding means and 95% credible intervals (CI), using eqn 7 and the MH algorithm described in Appendix S1. For this purpose, use pilot runs to tune up the proposal distributions to obtain acceptance rates between 40−50%, after which run the MH algorithm for 260,000 iterations, discarding the first 10,000 as burn-in and thinning the remaining ones to every 25^th^, yielding a final sample of 1,000 observations.
6. Given the two temporal genotypic samples of size *n*, conduct quasi-exact and Monte Carlo neutrality tests for each locus using, respectively, eqn 2 and the non-parametric approach described in the main text.

For each combination of assumed parameter values, the six simulation steps were repeated to generate *R* = 1,000 independent realizations of the process and their associated $\hat{\mathbf{\Theta}}$estimates, as well as neutrality test *p*-values. Expected estimation errors of the Bayesian estimation method were calculated by comparing $\hat{\mathbf{\Theta}}$ against $\mathbf{\Theta}$. As an example, the expected bias, expected root mean square error (*RMSE*), and expected CI non-coverage rate (NCR) of *s* were calculated with the following equations:

where $\hat{s}_{i}$ is the estimated selection coefficient for the *i*-th replicate data set, *s* is the assumed selection coefficient value and *A* is an indicator function taking the value 1 if the CI of $\hat{s}_{i}$ contains *s* or zero otherwise (i.e. *NCR* is the proportion of times that the 95% CI does not contain the assumed value).

For simulations assuming *s* = 0 (neutrality), the expected *type I error* (false positive) rate of each of the two neutrality tests were calculated as the proportion of simulated replicates where the corresponding test yielded a *p*-value smaller than the significance level α.

For simulations assuming *s* > 0, the expected *power* of each of the two neutrality tests was calculated as the proportion of simulated replicates where the corresponding test yielded a *p*-value smaller than α, and the *type II error* (false negative) rate as the proportion of simulated replicates where the test yielded a *p*-value larger than α.

**Figure S1.** Scatter plot of *p*-values obtained with the quasi-exact neutrality test and the Monte Carlo (MC) fast approximation. The dotted reference line has a slope equal to one. The simulated scenarios are the ones described in Figs. 1, 2 and 3 (main text) with *n* = 100 and *n* = 500.


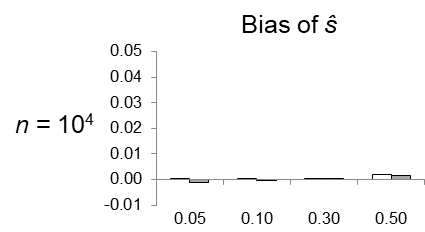

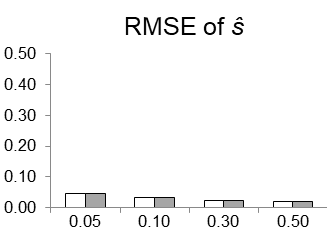

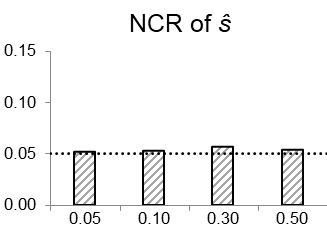

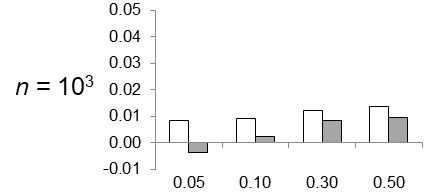

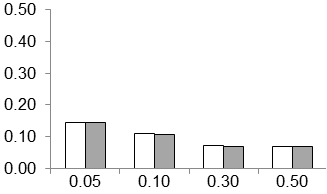

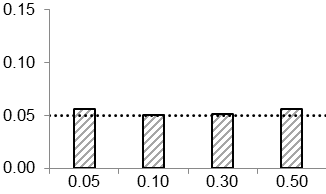

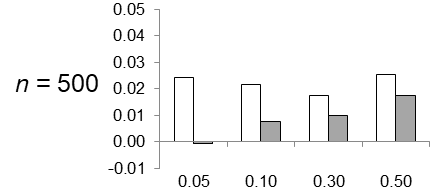

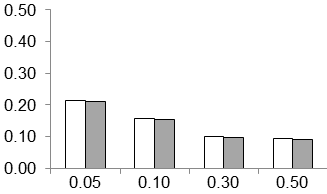

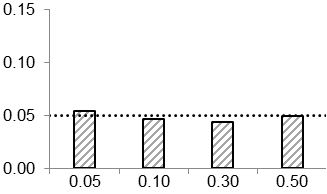

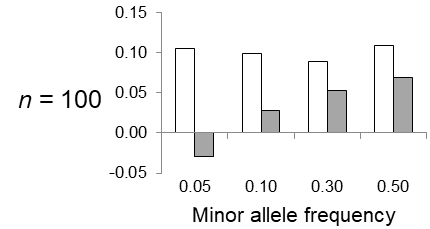

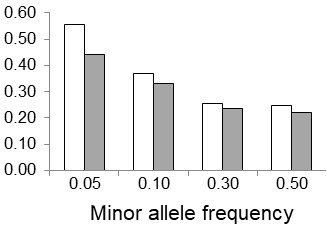

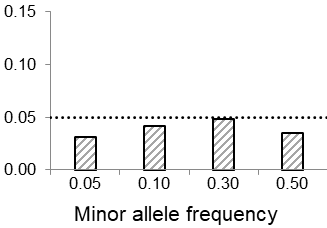


**Fig. S2**. Effect of sample size (*n*) and minor allele frequency on selection coefficient estimates for a neutral locus (*s* = 0). RMSE is the root mean square error and NCR the non-coverage rate of 95% credible intervals (the dotted line shows the nominal 5% value). Bias and RMSE were measured with respect to the mean (white bars) or with respect to the median (grey bars). Based on 1,000 Monte Carlo replicates per scenario, assuming a cohort of size *N* = 100,000 and a bi-allelic locus.


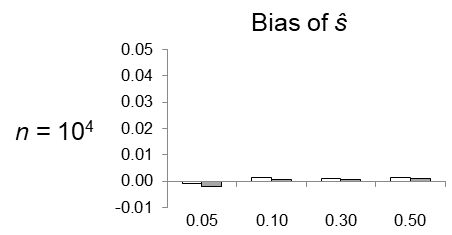

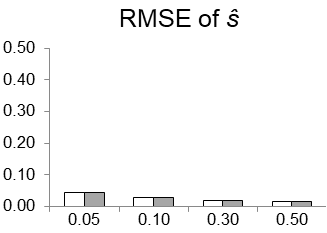

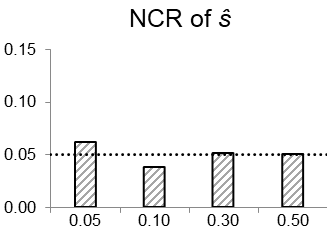

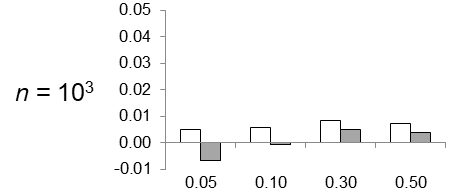

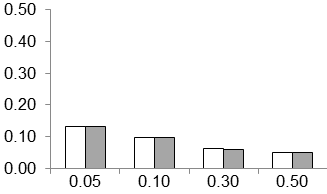

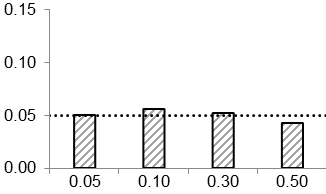

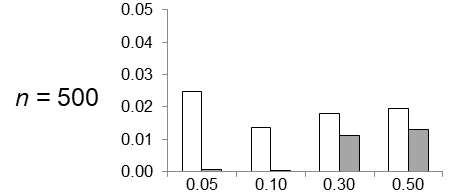

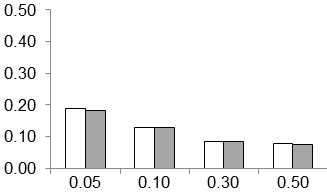

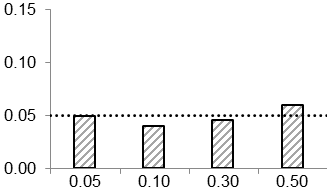

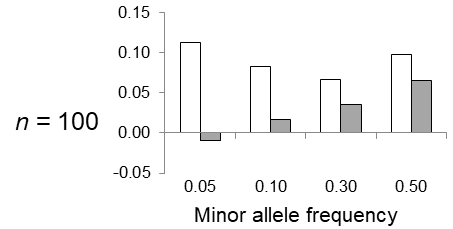

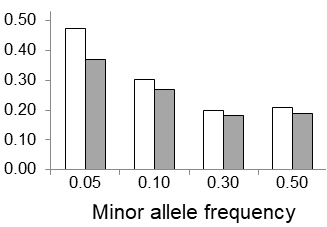

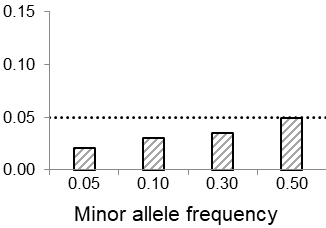


**Fig. S3**. Effect of sample size (*n*) and minor allele frequency on selection coefficient estimates for a locus under strong selection (*s* = −0.1). RMSE is the root mean square error and NCR the non-coverage rate of 95% credible intervals (the dotted line shows the nominal 5% value). Bias and RMSE were measured with respect to the mean (white bars) or with respect to the median (grey bars). Based on 1,000 Monte Carlo replicates per scenario, assuming a cohort of size *N*= 100,000 and a locus with two codominant alleles.


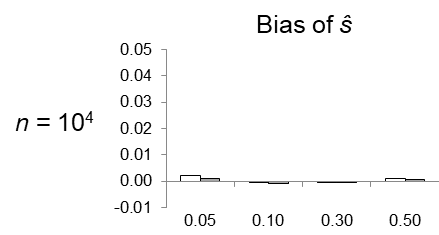

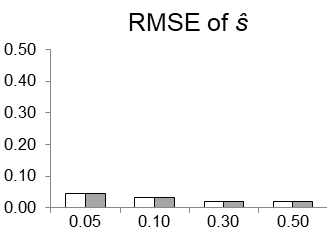

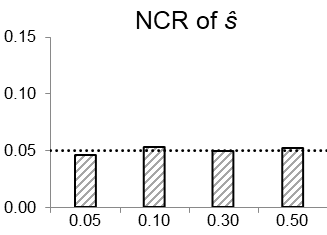

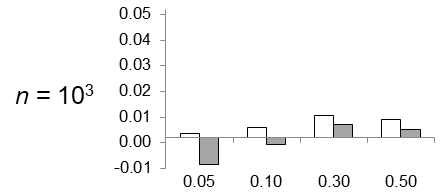

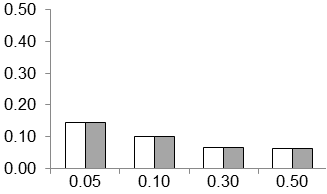

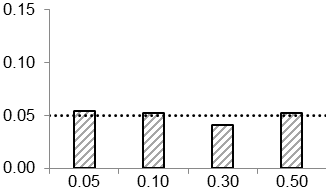

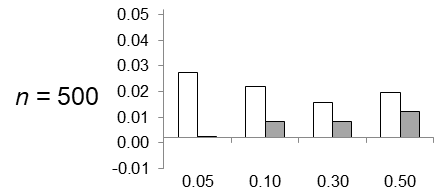

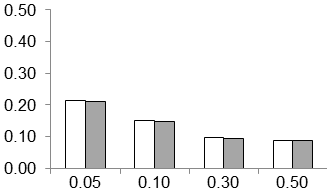

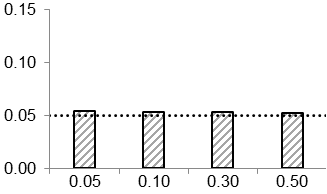

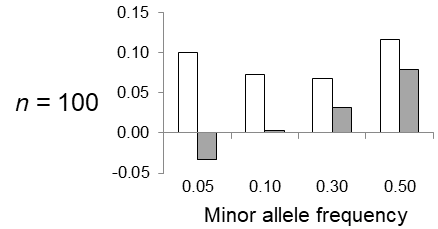

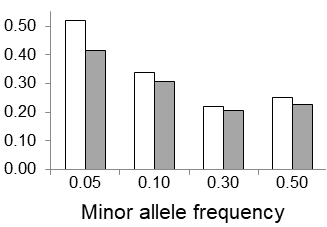

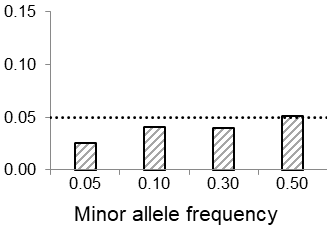


**Fig. S4**. Effect of sample size (*n*) and minor allele frequency on selection coefficient estimates for a locus under weak selection (*s* = −0.01). RMSE is the root mean square error and NCR the non-coverage rate of 95% credible intervals (the dotted line shows the nominal 5% value). Bias and RMSE were measured with respect to the mean (white bars) or with respect to the median (grey bars). Based on 1,000 Monte Carlo replicates per scenario, assuming a cohort of size *N*= 100,000 and a locus with two codominant alleles.


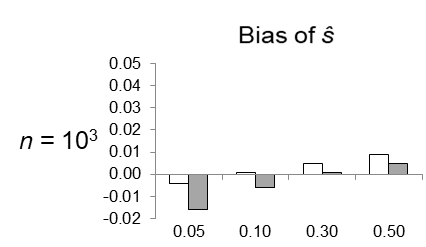

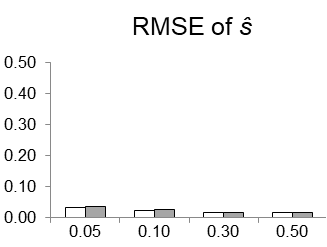

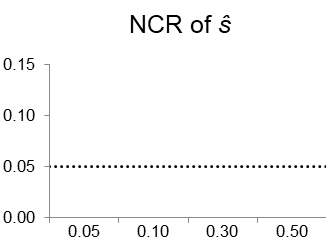

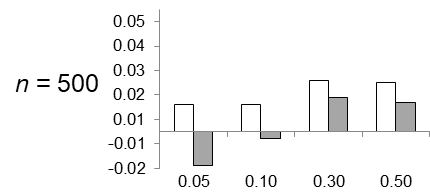

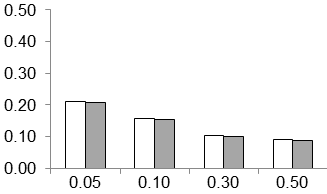

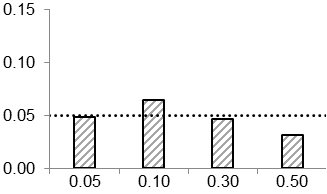

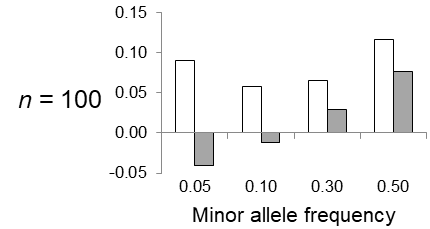

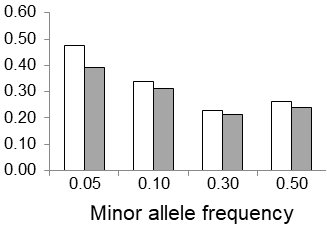

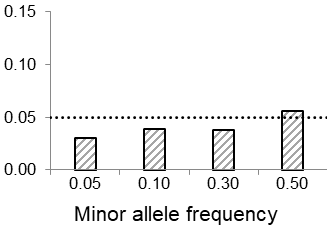


**Fig. S5**. Effect of sample size (*n*) and minor allele frequency on selection coefficient estimates for a neutral locus (*s* = 0). RMSE is the root mean square error and NCR the non-coverage rate of 95% credible intervals (the dotted line shows the nominal 5% value). Bias and RMSE were measured with respect to the mean (white bars) or with respect to the median (grey bars). Based on 1,000 Monte Carlo replicates per scenario, assuming a cohort of size *N* = 1,000 and a bi-allelic locus.


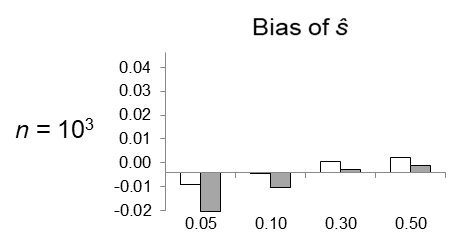

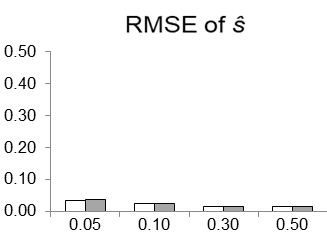

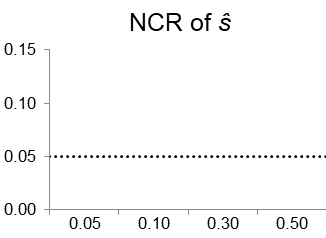

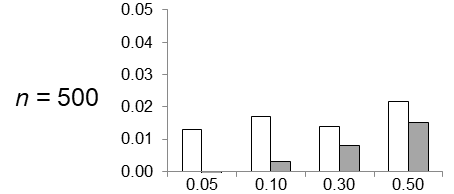

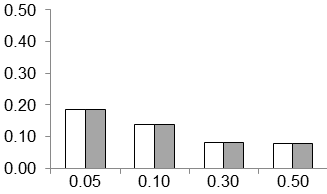

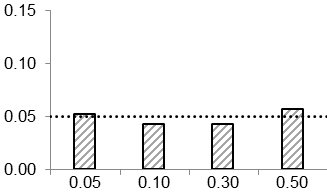

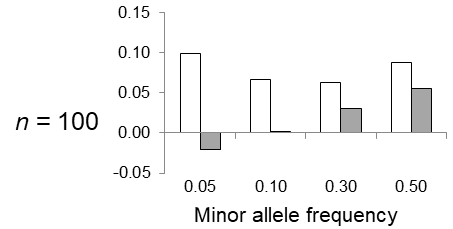

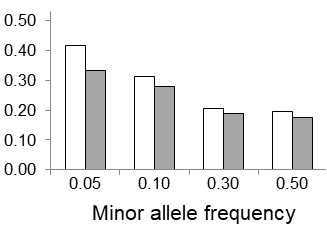

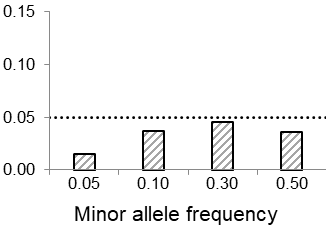


**Fig. S6**. Effect of sample size (*n*) and minor allele frequency on selection coefficient estimates for a locus under strong selection (*s* = −0.1). RMSE is the root mean square error and NCR the non-coverage rate of 95% credible intervals (the dotted line shows the nominal 5% value). Bias and RMSE were measured with respect to the mean (white bars) or with respect to the median (grey bars). Based on 1,000 Monte Carlo replicates per scenario, assuming a cohort of size *N*= 1,000 and a locus with two codominant alleles.


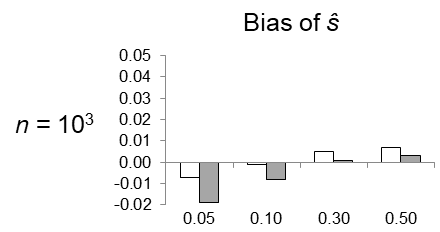

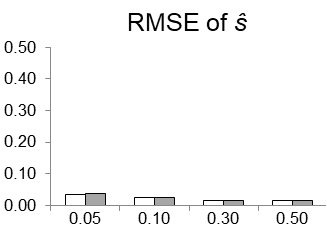

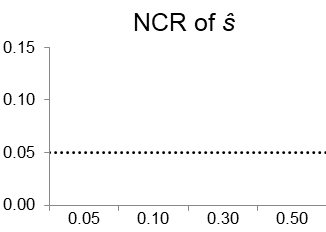

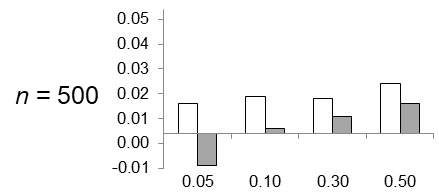

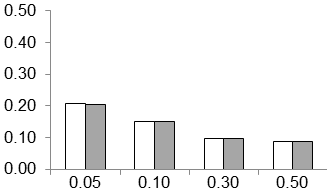

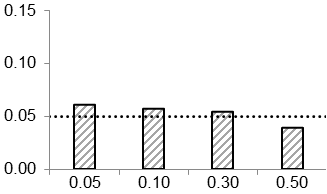

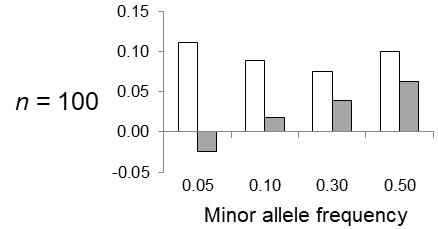

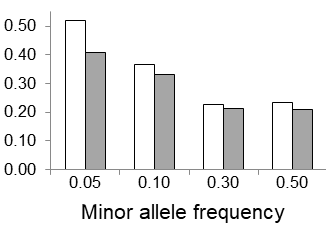

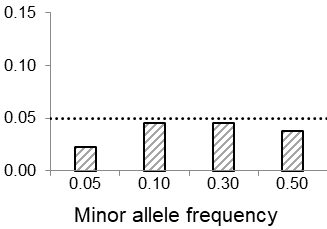


**Fig. S7**. Effect of sample size (*n*) and minor allele frequency on selection coefficient estimates for a locus under weak selection (*s* = −0.01). RMSE is the root mean square error and NCR the non-coverage rate of 95% credible intervals (the dotted line shows the nominal 5% value). Bias and RMSE were measured with respect to the mean (white bars) or with respect to the median (grey bars). Based on 1,000 Monte Carlo replicates per scenario, assuming a cohort of size *N*= 1,000 and a locus with two codominant alleles.


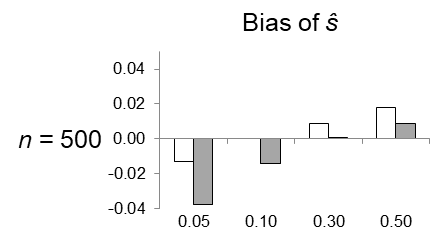

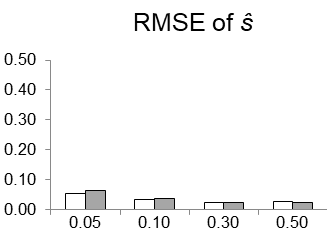

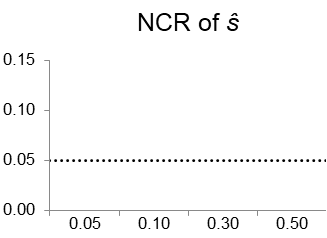

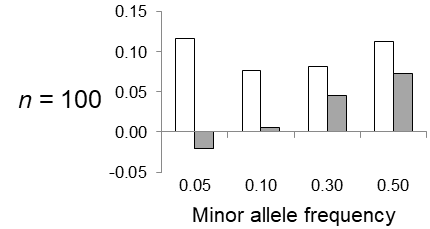

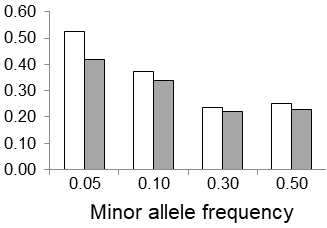

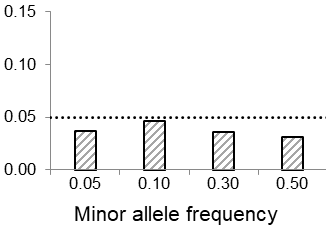


**Fig. S8**. Effect of sample size (*n*) and minor allele frequency on selection coefficient estimates for a neutral locus (*s* = 0). RMSE is the root mean square error and NCR the non-coverage rate of 95% credible intervals (the dotted line shows the nominal 5% value). Bias and RMSE were measured with respect to the mean (white bars) or with respect to the median (grey bars). Based on 1,000 Monte Carlo replicates per scenario, assuming a cohort of size *N* = 500 and a bi-allelic locus.


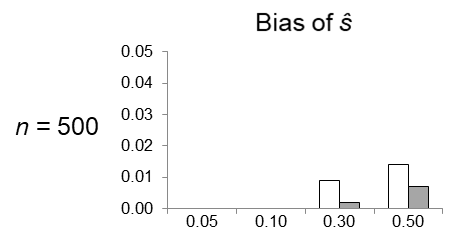

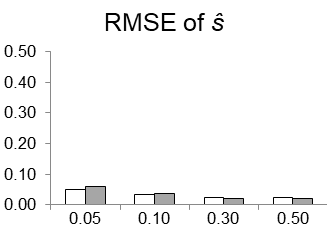

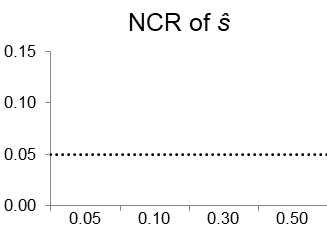

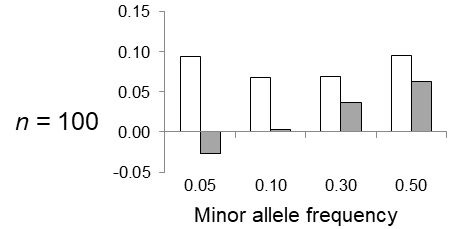

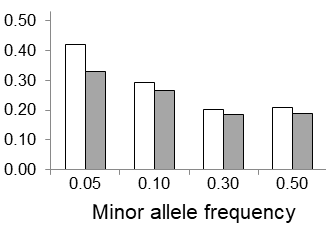

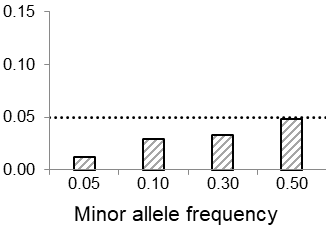


**Fig. S9**. Effect of sample size (*n*) and minor allele frequency on selection coefficient estimates for a locus under strong selection (*s* = −0.1). RMSE is the root mean square error and NCR the non-coverage rate of 95% credible intervals (the dotted line shows the nominal 5% value). Bias and RMSE were measured with respect to the mean (white bars) or with respect to the median (grey bars). Based on 1,000 Monte Carlo replicates per scenario, assuming a cohort of size *N*= 500 and a locus with two codominant alleles.


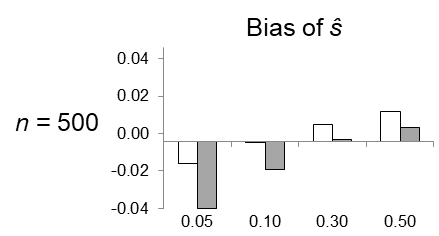

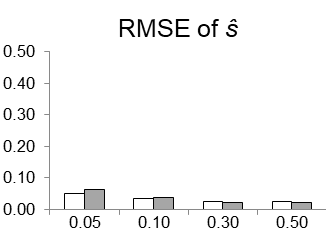

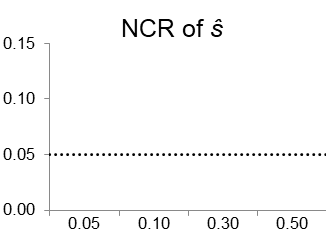

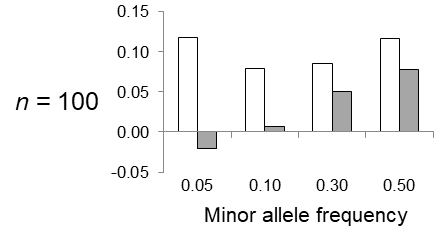

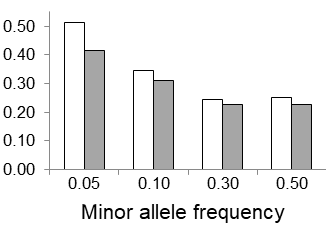

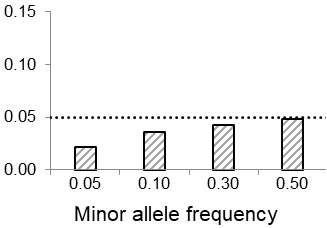


**Fig. S10**. Effect of sample size (*n*) and minor allele frequency on selection coefficient estimates for a locus under weak selection (*s* = −0.01). RMSE is the root mean square error and NCR the non-coverage rate of 95% credible intervals (the dotted line shows the nominal 5% value). Bias and RMSE were measured with respect to the mean (white bars) or with respect to the median (grey bars). Based on 1,000 Monte Carlo replicates per scenario, assuming a cohort of size *N =* 500 and a locus with two codominant alleles.


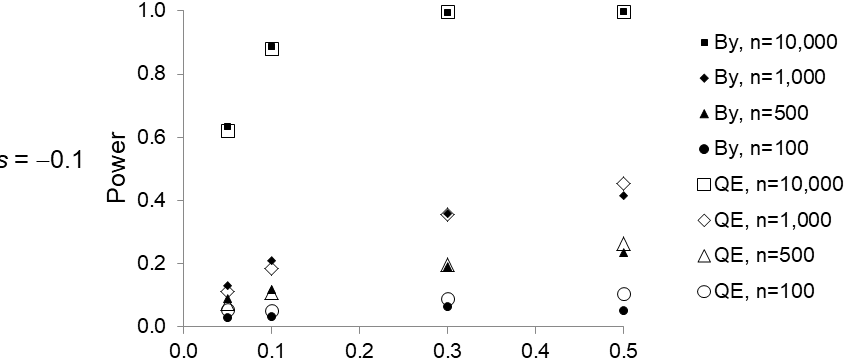

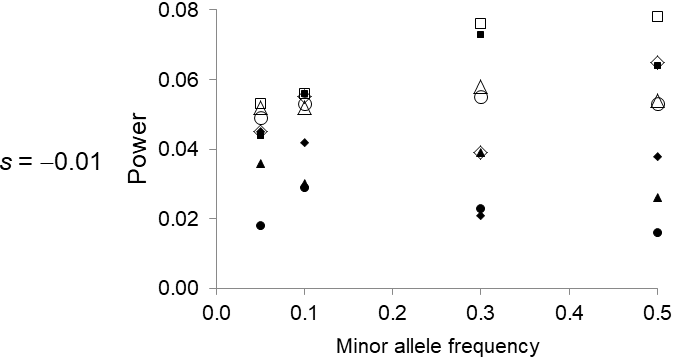

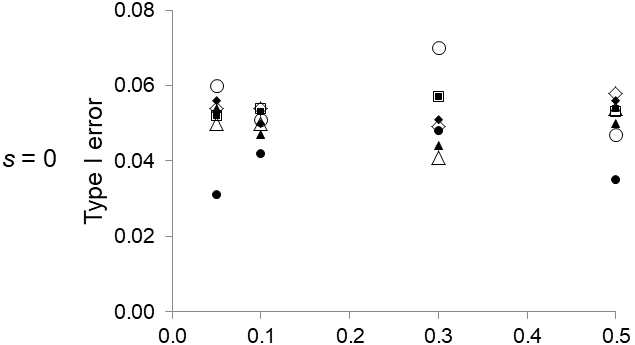


**Fig. S11**. Effect of sample size and minor allele frequency on the false positive (Type I error) rate and the power of neutrality tests. The Type I error was calculated assuming a neutral locus (*s* = 0; top panel), and the power assuming a strongly (*s* = −0.1; middle panel) or a weakly (*s* = −0.01; bottom panel) selected locus. The tests corresponded to either the quasi-exact neutrality test in eqn. 2 (“QE” white symbols) or to the proportion of times the 95% CI of Bayesian *s*-estimates did not include zero (“By” black symbols). Assumed temporal sample sizes were *n* = 100 (circles), *n* = 500 (triangles), *n* = 1,000 (diamonds) and *n* = 10,000 (squares). Based on 1,000 Monte Carlo replicates per scenario, assuming a cohort of size *N*= 100,000 and a locus with two codominant alleles.


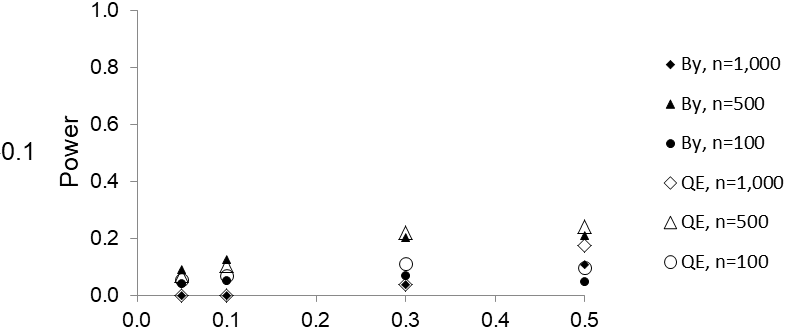

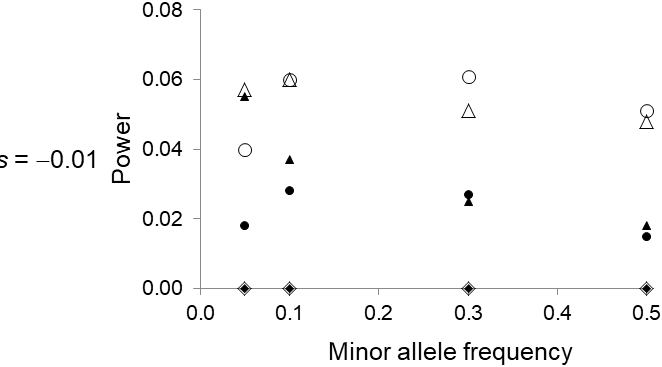

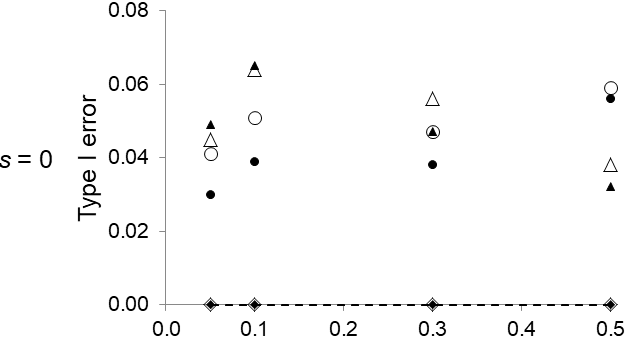


**Fig. S12**. Effect of sample size and minor allele frequency on the false positive (Type I error) rate and the power of neutrality tests. The Type I error was calculated assuming a neutral locus (*s* = 0; top panel), and the power assuming a strongly (*s* = −0.1; middle panel) or a weakly (*s* = −0.01; bottom panel) selected locus. The tests corresponded to either the quasi-exact neutrality test in eqn. 2 (“QE” white symbols) or to the proportion of times the 95% CI of Bayesian *s*-estimates did not include zero (“By” black symbols). Assumed temporal sample sizes were *n* = 100 (circles), *n* = 500 (triangles) and *n* = 1,000 (diamonds). Based on 1,000 Monte Carlo replicates per scenario, assuming a cohort of size *N*= 1,000 and a locus with two codominant alleles.


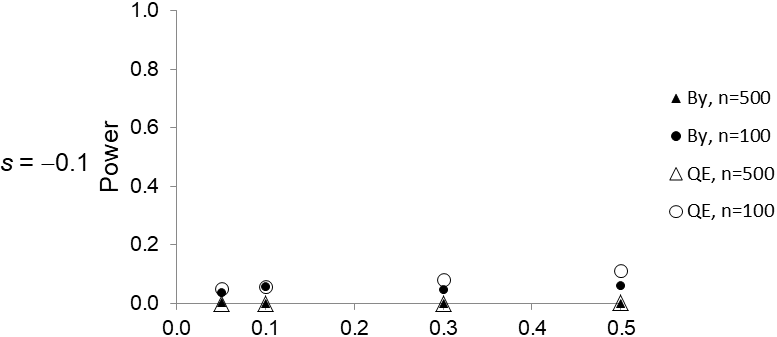

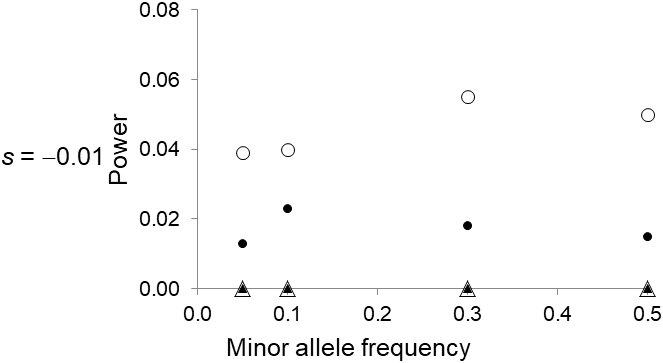

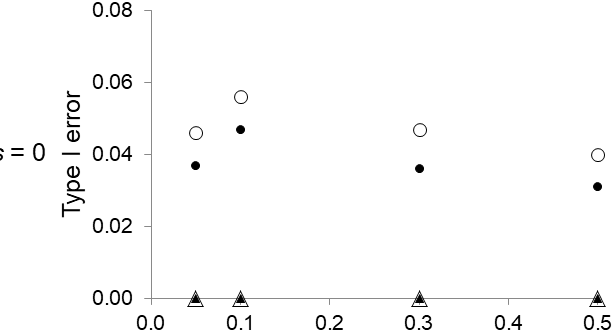


**Fig. S13**. Effect of sample size and minor allele frequency on the false positive (Type I error) rate and the power of neutrality tests. The Type I error was calculated assuming a neutral locus (*s* = 0; top panel), and the power assuming a strongly (*s* = −0.1; middle panel) or a weakly (*s* = −0.01; bottom panel) selected locus. The tests corresponded to either the quasi-exact neutrality test in eqn. 2 (“QE” white symbols) or to the proportion of times the 95% CI of Bayesian *s*-estimates did not include zero (“By” black symbols). Assumed temporal sample sizes were *n* = 100 (circles) and *n* = 500 (triangles). Based on 1,000 Monte Carlo replicates per scenario, assuming a cohort of size *N*= 500 and a locus with two codominant alleles
